# Transcriptomic profiling of the myeloma bone-lining niche reveals BMP signalling inhibition to improve bone disease

**DOI:** 10.1038/s41467-019-12296-1

**Published:** 2019-10-04

**Authors:** Sarah Gooding, Sam W. Z. Olechnowicz, Emma V. Morris, Andrew E. Armitage, Joao Arezes, Joe Frost, Emmanouela Repapi, James R. Edwards, Neil Ashley, Craig Waugh, Nicola Gray, Erik Martinez-Hackert, Pei Jin Lim, Sant-Rayn Pasricha, Helen Knowles, Adam J. Mead, Karthik Ramasamy, Hal Drakesmith, Claire M. Edwards

**Affiliations:** 1 MRC Human Immunology Unit, MRC Weatherall Institute of Molecular Medicine, University of Oxford, Oxford, UK; 2 Oxford University Hospitals NHS Trust, Oxford, UK; 3 NIHR Oxford Biomedical Research Centre Blood Theme, University of Oxford, Oxford, UK; 4 Oxford Centre for Translational Myeloma Research, University of Oxford, Oxford, UK; 5 Nuffield Dept. of Surgical Sciences, University of Oxford, Oxford, UK; 6 MRC Weatherall Institute of Molecular Medicine, University of Oxford, Oxford, UK; 7 Nuffield Dept. of Orthopaedics, Rheumatology and Musculoskeletal Sciences, Botnar Research Centre, University of Oxford, Oxford, UK; 8 Department of Biochemistry and Molecular Biology, Michigan State University, East Lansing, MI 48824 USA

**Keywords:** Cancer microenvironment, Bone, Myeloma

## Abstract

Multiple myeloma is an incurable, bone marrow-dwelling malignancy that disrupts bone homeostasis causing skeletal damage and pain. Mechanisms underlying myeloma-induced bone destruction are poorly understood and current therapies do not restore lost bone mass. Using transcriptomic profiling of isolated bone lining cell subtypes from a murine myeloma model, we find that bone morphogenetic protein (BMP) signalling is upregulated in stromal progenitor cells. BMP signalling has not previously been reported to be dysregulated in myeloma bone disease. Inhibition of BMP signalling in vivo using either a small molecule BMP receptor antagonist or a solubilized BMPR1a-FC receptor ligand trap prevents trabecular and cortical bone volume loss caused by myeloma, without increasing tumour burden. BMP inhibition directly reduces osteoclastogenesis, increases osteoblasts and bone formation, and suppresses bone marrow sclerostin levels. In summary we describe a novel role for the BMP pathway in myeloma-induced bone disease that can be therapeutically targeted.

## Abstract (web summary)

Multiple myeloma is a cancer of the bone marrow that can induce bone disease. Here, the authors profile the transcriptome of bone-lining cells and find a targetable role of bone morphogenetic protein (BMP) signalling in myeloma-induced bone-disease

## Introduction

Multiple myeloma is an incurable malignancy of terminally differentiated plasma cells with a remarkable ability to manipulate the bone marrow environment^1^. Increased and uncoupled local bone remodelling results in the characteristic osteolytic bone disease, typified by destruction of large areas of mature bone. These lesions frequently cause bone pain, and lead to complications, including pathological fractures, vertebral collapse, cord compression, hypercalcaemia and generalised osteoporosis^2^. Myeloma patients may present with bone disease at diagnosis or develop it during the course of their illness, and it is standard practice to treat with bisphosphonates or similar anti-resorptive therapies to minimise further bone loss. However, no current therapy can restore pre-existing bone loss. Furthermore at disease relapse, bone pain may return and osteolytic lesions progress. There is hence an urgent need to develop new approaches to combat myeloma bone disease.

In adult bone homeostasis, endosteal surfaces are lined with quiescent ‘bone-lining cells’^3^ (BLCs), originating from skeletal stem cells, which become activated when bone remodelling is required, to become matrix-secreting osteoblasts^4,5^. However, in the presence of active myeloma, this BLC layer is disrupted, with impaired osteoblast differentiation and function resulting in an inability to repair damage. An expansion in osteoclast number and activity, coupled to maturation arrest and reduction in osteoblast numbers, tips the normal balance between bone loss and bone formation decisively towards loss^2^. Various signalling pathways are implicated in the osteoblast differentiation block, including activin A, tumour growth factor-β (TGFβ), Notch and Wnt pathways^6^. Wnt inhibitors Dkk1 and sclerostin are elevated in the serum and bone marrow of patients with myeloma, and blocking their action improves myeloma bone disease pre-clinically^7–9^.

With increasing numbers of myeloma tumour genomes sequenced, substantial knowledge has been gained regarding genetic changes in myeloma cells^10^. However, the transcriptional changes that occur in the bone marrow niche compartments surrounding and supporting myeloma in vivo are unknown. Since alterations in the myeloma niche are key in supporting tumour survival, the changes that occur when myeloma develops must be better understood in order to identify new therapeutic approaches to manage bone pathology.

Based on the hypothesis that myeloma invasion would induce changes in niche gene expression profiles, we performed BLC transcriptome profiling in an in vivo myeloma murine model. Our analysis revealed upregulation of the bone morphogenetic protein (BMP) signalling pathway in mesenchymal stromal progenitor cells in myeloma-bearing mice. BMPs have been shown to have directly toxic effects on myeloma cells in vitro^11–13^; here we show that experimental inhibition of this signalling pathway in multiple myeloma models restored bone mass without any adverse effect on tumour bulk, revealing a novel role for BMP signalling in myeloma bone disease.

## Results

### BMP signalling is altered in the myeloma bone-lining niche

**Fig. 1:**
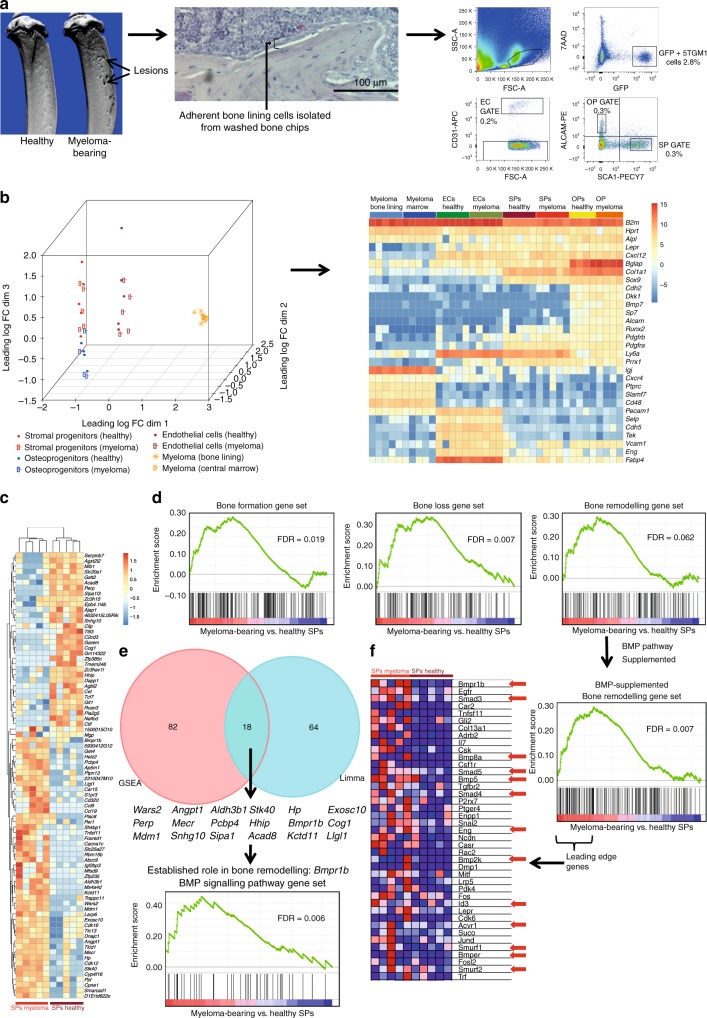
Transcriptome profiling of bone-lining niche components from myeloma-bearing mice identifies dysregulated BMP signalling. **a** Bone-lining cells disassociated from the bone surface of hindlimb long bones using enzyme digestion were FACS-sorted for RNA-Seq in 100 cell aliquots. Percentages shown are a proportion of the live cell gate. GFP+ cell abundance varied with tumour burden. The full gating strategy is shown in Supplementary Fig. 1. **b** Multidimensional Scaling (MDS) plot of the four cell types sorted, using leading log-fold changes in gene expression between each pair of samples. To confirm cell phenotypes, a heat map was generated using established marker gene expression for each cell type (colour scale; log RPKM values). **c** Heat map of stromal progenitor gene expression, showing genes differentially expressed with adj. *p* value <0.1 and abs(log 2FC) >1 (*n* = 82) (colour scale; centred and scaled log RPKM values). **d** Myeloma-bearing vs. healthy stromal progenitor GSEA enrichment plots for bone formation (FDR 0.019), bone loss (FDR 0.007) and bone remodelling (FDR 0.062) gene sets. **e** Overlap between limma-generated differentially expressed genes (adj. *p* value <0.1 and abs(log 2FC) >1) and GSEA-ranked top 100 genes (*n* = 18). Only *Bmpr1b* has established role in bone remodelling. GSEA of BMP signalling gene set showed enrichment in myeloma-bearing vs. healthy stromal progenitors (FDR 0.006). **f** The bone remodelling gene set (*n* = 151) supplemented with BMP family members (*n* = 44, 21%) and reanalysed: FDR decreased from 0.062 to 0.007. The gene set leading edge is shown, comprising 32% BMP pathway members, targets or interactors (marked with red arrows). Colour scale as per GSEA algorithm: the range of colours (red, pink, light blue, dark blue) shows the range of RPKM expression values (high, moderate, low, lowest). OP: osteoprogenitors; SP: stromal progenitors; EC: endothelial cells

To examine how myeloma alters gene expression within the bone-lining niche in vivo, we isolated and performed RNA-sequencing (RNA-Seq) on distinct cell populations from the bones of mice previously inoculated with 5TGM1-GFP (green fluorescent protein) myeloma cells, or non-tumour controls. This model was selected because of its recapitulation of human myeloma bone disease^14^. Using an approach to isolate endosteal niche populations reported by Nakamura et al.^15^, we sorted osteoprogenitors (ALCAM+Sca1−), stromal progenitor cells (Sca1+ALCAM−^15,16^), and endothelial cells (CD31+) from the bone-lining, and GFP+ myeloma cells from the bone-lining and central bone marrow using multi-colour fluorescence-activated cell sorting (Fig. 1a and Supplementary Fig. 1). Gene expression analysis with multidimensional scaling (MDS) demonstrated separation of cell types (Fig. 1b). A heat map was generated using marker gene expression for each cell type (Supplementary Table 1), confirming cell-type-specific expression profiles (Fig. 1b and Supplementary Fig. 2). Stromal progenitors and osteoprogenitors underwent further characterisation; significant increases in alkaline phosphatase, *osteocalcin* and *osterix* were detected in sorted osteoblasts as compared to stromal progenitors. Differentiation studies of isolated stromal progenitor and osteoprogenitor populations from healthy mice demonstrated the multi-lineage potential of stromal progenitors to differentiate into osteoblasts and adipocytes, as compared to the restricted differentiation of sorted osteoprogenitors (Supplementary Figs. 3–5).

We assessed differential gene expression using the voom-limma method^17–19^, with significance criteria of adjusted *p* value < 0.1 and abs(log 2FC) >1. We found only 6 differentially expressed genes between myeloma cells from the two niches (bone-lining vs. central bone marrow), and 251 differing between endothelial cells from myeloma-bearing and control mice. There were greater transcriptome differences in stromal progenitor (82 genes) than osteoprogenitor (28 genes) subsets. We screened endothelial, stromal progenitor, and osteoprogenitor gene expression data using gene set enrichment analysis (GSEA) MSigDB Hallmark gene sets^20^, revealing common upregulation of interferon response sets in all cell types from myeloma-bearing mice, and cell cycle-related sets in stromal progenitors and endothelial cells (Supplementary Fig. 6). The greatest number of upregulated sets was in stromal progenitors. We proceeded to use GSEA to analyse differentially expressed genes in stromal progenitors to investigate whether any bone therapeutic targets were implicated (Fig. 1c). We pre-designed three gene sets to examine changes in bone homeostasis, designated ‘bone formation’, ‘bone loss’ and ‘bone remodelling’ (Supplementary Data 1). Although the ‘bone remodelling’ set (false discovery rate (FDR) 0.062) did not reach significance, the ‘bone loss’ and ‘bone formation’ gene sets were significantly enriched in bone-lining stromal progenitors from myeloma-bearing mice compared to controls (Fig. 1d).

We next assessed the overlap between limma-generated differentially expressed genes and GSEA enrichment score-ranked top 100 genes in stromal progenitors. This identified 18 common genes (Fig. 1e). Of these, only *Bmpr1b* is an established regulator of bone homeostasis^21^. BMP signalling has roles in both bone formation and resorption^22^, but the gene set combining these processes, bone remodelling, was found to contain only three BMP pathway genes, *Acvr1*, *Bmp4* and *Grem1*. Having identified *Bmpr1b* to be differentially expressed, we hypothesised that other members of the BMP pathway may also be differentially expressed. We therefore designed a BMP signalling pathway gene set (Supplementary Data 1), which we found to be significantly enriched in stromal progenitors from myeloma-bearing mice (Fig. 1e). To assess if this enrichment was equivalent in magnitude to that of our other bone homeostasis sets, we expanded the bone remodelling gene set to include the BMP pathway gene set members (*n* = 44). In this expanded gene set, 13/41 of the leading edge genes driving enrichment were BMP gene set members, supporting a role for the BMP pathway in the disordered bone homeostasis of myeloma bone disease (Fig. 1f).

### BMP signalling blockade reduces myeloma bone disease in vivo

**Fig. 2:**
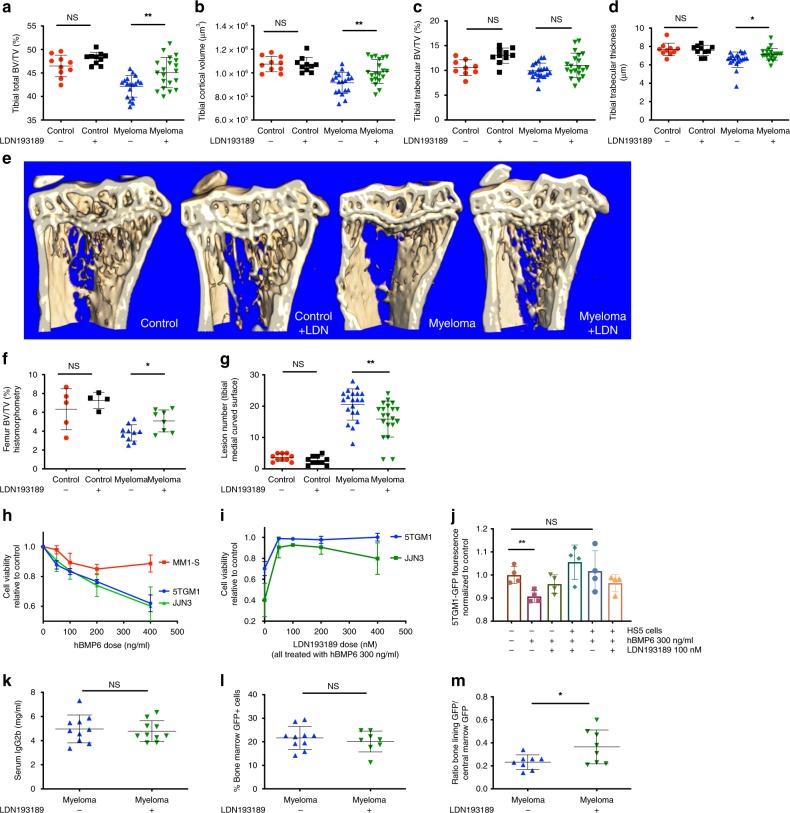
Inhibition of BMP signalling with LDN193189 reduces myeloma bone disease in vivo, with no effect on tumour burden. *n* = 10 per control group, 20 per myeloma group, total *n* = 60 for microCT-derived indices; *n* = 5 per control group, 10 per myeloma group, total *n* = 30 for other indices. Missing data values: see Methods. *P < 0.05, **p < 0.01, ***p < 0.001, NS, not significant, *t* test. Source data are provided as a Source Data file. Reconstructed tibial metaphyseal images were analysed using Bruker CTAn software to generate (**a**–**d**): **a** total BV/TV (bone volume/total volume). **b** Cortical volume in μm^3^. **c** Trabecular BV/TV. **d** Trabecular thickness in μm. **e** Reconstructed microCT images of tibial sagittal sections showing trabecular architecture. **f** Histomorphometric generated BV/TV (femoral metaphyseal sections). **g** Enumerated cortical lytic lesions on right medial tibial surface. **h** Effect of BMP6 on JJN-3, MM1-S (human) and 5TGM1 (mouse) myeloma cell line viability at 72 h (alamarBlue® assay), *n* = 4 replicates. Source data are provided as a Source Data file. **i** Dose effect of LDN193189 on BMP6-induced myeloma growth inhibition at 72 h. Viability was expressed relative to viability in control conditions, *n* = 3 replicates. Source data are provided as a Source Data file. **j** Effect of hBMP6, LDN193189 and contact co-culture with stromal line HS5 on proliferation of 5TGM1-GFP cells (GFP fluorescence intensity as proxy for growth), *n* = 4 replicates. **k** 5TGM1-GFP myeloma disease burden quantification: serum IgG2b level (mg/ml). **l** 5TGM1-GFP myeloma disease burden quantification: bone marrow GFP+ percentage. **m** Effect of LDN193189 on myeloma distribution between central marrow niche (flushed from marrow cavity) and bone-lining niche (collagenase-digested from cleaned bone fragment surfaces) (*n* = 8 per group). Data represent mean ± SD

The enriched expression of BMP pathway members in stromal progenitors from myeloma-bearing mice suggests that this pathway may influence myeloma bone disease pathogenesis. To test this, we treated 5TGM1 myeloma-bearing mice and controls with LDN193189 (LDN), a small-molecule inhibitor that functions as an antagonist of BMP type 1 receptors. LDN has been well characterised in vitro and in vivo, with high specificity for type 1 BMP receptor signalling as compared to TGFβ or activin-mediated SMAD2/3 signalling^23^. LDN treatment abrogated BMP6-induced SMAD1/5/9 phosphorylation in 2T3 preosteoblasts in vitro (Supplementary Fig. 7). Commensurate with human myeloma bone disease, myeloma-bearing mice had reduced trabecular and cortical bone volume and increased osteolytic bone lesions. Treatment with LDN increased cortical and trabecular bone volume, trabecular thickness and decreased osteolytic lesions in myeloma-bearing mice (Fig. 2a–g).

Direct anti-tumour effects of BMPs against myeloma have been described in vitro^11–13^. Consistent with this, we found treatment of human JJN-3 and murine 5TGM1 myeloma cells with recombinant human BMP6 induced a dose-dependent reduction in myeloma cell viability (Fig. 2h) that was rescued in both cell lines by treatment with LDN (Fig. 2i). However, BMP6-mediated inhibition of 5TGM1 myeloma cell growth was also prevented by co-culture with HS5 bone marrow stromal cells in the absence of LDN (Fig. 2j), suggesting that the microenvironment may also mediate the response of myeloma cells to BMP signalling. It was therefore important to determine the effect of BMP inhibition on myeloma tumour burden in vivo. LDN had no effect on overall tumour burden in vivo, as measured by myeloma-specific serum IgG2bk concentrations (Fig. 2k). Similarly, we observed no significant difference in the proportion of myeloma cells within the central marrow (Fig. 2l). Interestingly, there was a significant increase in the proportion of myeloma cells adherent to the bone-lining niche following LDN treatment (Fig. 2m). However, there were no significant gene set enrichment differences in bone-adherent myeloma cells between LDN and vehicle-treated mice to indicate phenotypic changes accompanying their increased proportion (Hallmark and Reactome gene sets interrogated^20,24^, Supplementary Table 2). When bulk, non-adherent central marrow myeloma cells from LDN and vehicle-treated mice were compared, a small number of gene sets were downregulated in myeloma cells from LDN-treated mice. None of these had direct relevance to cell proliferation, but included EMT, angiogenesis and those related to extracellular matrix interactions (Hallmark and Reactome gene sets interrogated, Supplementary Table 2).

**Fig. 3:**
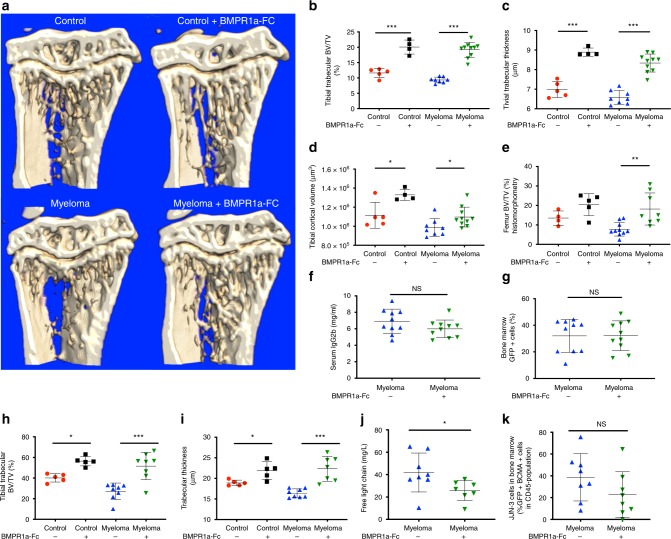
Inhibition of BMP signalling with BMPR1a-FC reduces myeloma bone disease in vivo, with no effect on tumour burden (5TGM1 model; *n* = 5 per control group, 10 per myeloma group, total *n* = 30. JJN-3 model; *n* = 5 per control group, 8 per myeloma group, total *n* = 26). *P < 0.05, **p < 0.01, ***p < 0.001, NS, not significant, *t* test. **a** Reconstructed microCT images of tibial sagittal sections showing trabecular architecture. Reconstructed tibial metaphyseal images analysed using Bruker CTAn software to generate **b**–**d**: **b** trabecular BV/TV (bone volume/total volume). **c** Trabecular thickness in μm. **d** Cortical volume in μm^3^. **e** Histomorphometric analysis of right femoral metaphysis sections using Osteomeasure software to generate BV/TV. **f** 5TGM1-GFP myeloma disease burden quantification using serum IgG2b level (mg/ml). **g** 5TGM1-GFP myeloma disease burden quantification using bone marrow GFP+ percentage. **h** Trabecular BV/TV (bone volume/total volume) in JJN-3 model. **i** Trabecular thickness in μm in JJN-3 model. **j** JJN-3 myeloma disease burden quantification using kappa free light chain quantification (mg/l). **k** JJN-3 bone marrow tumour burden was calculated by the sum of GFP+ and human BCMA+ cells as a proportion of murine CD45-negative population. Data represent mean ± SD

To extend our investigation into the role of BMPs in myeloma, we next used a BMPR1a-Fc-solubilised ligand trap, which binds and neutralises BMP ligands that bind to the Alk3 (BMPR1a) receptor, including BMPs 2, 4, 5, 6, 7 and 8^25^. Basal expression of *Bmpr1a* was higher than *Bmpr1b* in stromal progenitors and osteoprogenitors, although *Bmpr1b* expression was significantly increased in stromal progenitors from myeloma-bearing mice (Supplementary Fig. 8). BMPR1a-Fc has previously been shown to increase bone volume in a murine model of osteoporosis^26^, but has never been investigated within the tumour–bone environment and represents a complementary approach to target the BMP pathway in myeloma bone disease in vivo. Myeloma-bearing mice had a significant increase in osteolytic bone lesions as determined by microCT (non-tumour: 3 ± 1; myeloma: 24.11 ± 5.278, *p* < 0.0001, unpaired *t* test). Treatment with BMPR1a-Fc completely prevented myeloma-associated trabecular bone volume loss (Fig. 3a–c). BMPR1a-Fc augmented trabecular and cortical bone parameters in non-tumour and myeloma-bearing mice (Fig. 3b–e), but had no effect on the number of lytic bone lesions (control: 24.11 ± 5.278; BMPR1a-Fc: 23.90 ± 5.384, mean ± SD). Consistent with LDN treatment, BMPR1a-Fc had no effect on tumour burden, and no effect on tumour cell apoptosis (Fig. 3f, g; Supplementary Fig. 9). We also demonstrated that BMP blockade was effective in reducing myeloma bone disease in a second in vivo model, the JJN-3 murine model of myeloma that uses human myeloma cells. BMPR1a-Fc treatment was found to significantly increase trabecular bone volume and trabecular thickness in both non-tumour and myeloma-bearing mice (Fig. 3h, i). BMPR1a-Fc significantly reduced overall tumour burden, as measured by serum free light chain quantification, but had no effect on the proportion of myeloma cells within the central marrow (Fig. 3j, k).

### BMP6 blockade does not reduce myeloma tumour or bone disease

BMP6 is the only BMP reported to be expressed by malignant and normal plasma cells in myeloma^27^. In support of this, 5TGM1 myeloma cells were found to express *Bmp6*, but not *Bmp2* or *Bmp4*, with *Bmp6* expression elevated by co-culture with 2T3 preosteoblasts (Supplementary Fig. 10). We therefore hypothesised that BMP6 may be responsible for the stromal progenitor BMP pathway upregulation we observed. To investigate this, 5TGM1 myeloma-bearing mice were treated with a neutralising anti-BMP6 antibody. In contrast to LDN and BMPR1a-FC, treatment of myeloma-bearing mice with anti-BMP6 had no effect on cortical or trabecular bone volume (Supplementary Fig. 11A, B). Blockade of BMP6 had no effect on overall tumour burden, nor the proportion of myeloma cells within the central bone marrow (Supplementary Fig. 11C, D). However, BMP6 is known to influence iron metabolism by inducing hepatic expression of the iron regulatory protein hepcidin, whose activity leads to reduced serum iron and, if chronically elevated, anaemia. Consistent with this, liver hepcidin (*Hamp1*) expression and serum hepcidin concentrations were reduced in anti-BMP6-treated myeloma-bearing mice, and serum iron and haemoglobin concentrations were increased (Supplementary Fig. 12). Taken together, these suggest that while BMP6 is unlikely to be a major mediator of myeloma bone disease, it may contribute to the anaemia associated with multiple myeloma. When we compared these results with the effects of LDN and BMPR1a-FC, we found that although both suppressed hepcidin (*HAMP*) gene expression by HuH7 cells in vitro (Supplementary Fig. 13), neither treatment reduced serum hepcidin in 5TGM1 myeloma-bearing mice. LDN reduced hepatic hepcidin gene expression in myeloma-bearing mice, but decreased serum hepcidin only in non-tumour mice (Supplementary Fig. 14). With BMPR1a-FC treatment, no in vivo changes in hepcidin gene expression or serum concentrations of hepcidin were observed in the 5TGM1 murine model of myeloma (Supplementary Fig. 15). In the JJN-3 model of myeloma, however, treatment with BMPR1a-Fc resulted in an increase in serum haemoglobin, concomitant with a reduction in hepatic hepcidin gene expression (Supplementary Fig. 15).

### BMP signalling blockade reduces osteoclast number

**Fig. 4:**
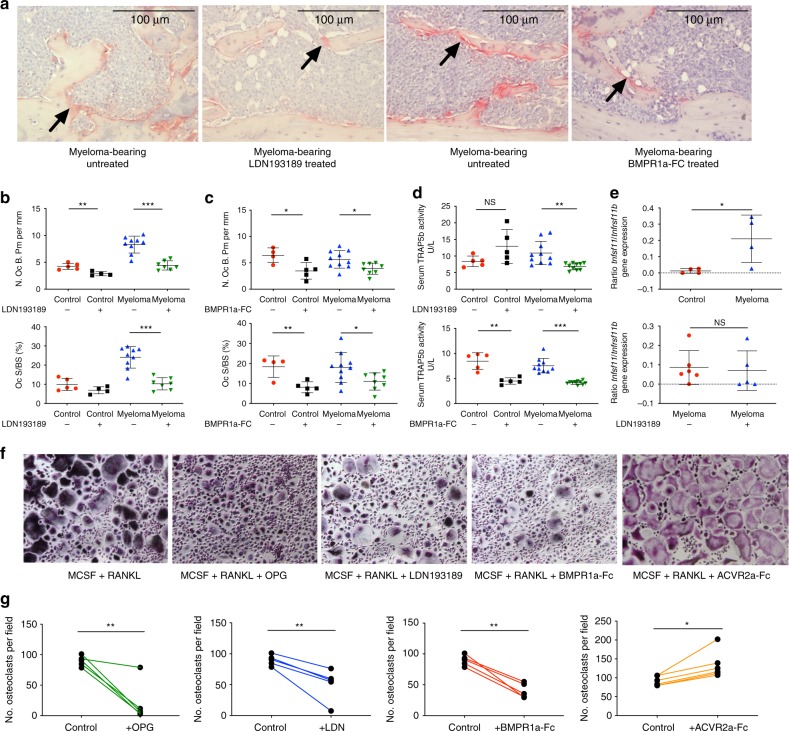
Inhibition of BMP signalling reduces osteoclast number in vivo and in vitro. **a** Osteoclasts (indicated with arrows) from myeloma-bearing mice: vehicle vs. LDN193189 treated and vehicle vs. BMPR1a-FC treated (TRAP and haematoxylin-stained). Scale bar = 100 μm. **b**, **c** Histomorphometric analysis of TRAP-stained osteoclast bone surface density (N.Oc.B.Pm) and osteoclast surface/ trabecular surface ratio (OcS/BS) in healthy and myeloma-bearing mice. *n* = 5 per control group, 10 per myeloma group and total *n* = 30. Missing data values: see Methods. **b** Vehicle vs. LDN193189 treated. **c** Vehicle vs. BMPR1a-FC treated. **d** Serum TRAP-5b concentration, indicating global osteoclast burden, in vehicle vs. LDN193189-treated and vehicle vs. BMPR1a-FC treated healthy and myeloma-bearing mice. **e** RNA-Seq-generated in vivo *Tnfsf11*:*Tnfrsf11b* (RANKL to OPG) expression in osteoblasts. Ratio in healthy vs. myeloma-bearing mice and vehicle vs. LDN193189-treated myeloma-bearing mice. **f** Pooled human CD14+ PBMC osteoclast differentiation, with treatments as indicated commenced 24 h after seeding, and TRAP-stained at day 10. Images were taken at ×70 magnification. **g** Quantified osteoclasts (≥3 nuclei) when CD14+ PBMCs treated as **f** were enumerated. Results from same experimental replicate are paired to account for inter-replicate variation (*n* = 5 replicates, paired *t* test). *P < 0.05, **p < 0.01, ***p < 0.001, NS, not significant, *t* test. Data represent mean ± SD

Since inhibition of BMP signalling, either by a receptor antagonist or a ligand trap, results in a significant reduction in myeloma bone disease in vivo, we further investigated the mechanisms underlying this bone-protective effect. Treatment with either LDN or BMPR1a-Fc led to a significant reduction in osteoclast number and the proportion of bone surface occupied by osteoclasts in myeloma-bearing mice, with osteoclast number also reduced in non-tumour-bearing animals (Fig. 4a–c). Serum TRAP (tartrate-resistant acid phosphatase) activity was reduced in myeloma-bearing mice with both compounds, indicative of reduced osteoclastic bone resorption (Fig. 4d; Supplementary Fig. 16). The receptor activator of nuclear factor κB ligand (RANKL) (*Tnfsf11*) to osteoprotegerin (OPG) (*Tnfrsf11b*) ratio was increased in osteoblasts isolated from myeloma-bearing mice as compared to control; however, treatment of myeloma-bearing mice with LDN had no effect on the RANKL to OPG ratio (Fig. 4e), suggesting that the reduced osteoclast burden was not due to RANKL/OPG-mediated changes.

We next investigated whether a direct effect of LDN or BMPR1a-FC on osteoclastogenesis could account for the reduced osteoclasts observed in vivo. Both treatments reduced osteoclast formation in vitro to an extent similar to OPG (Fig. 4f, g). Activin inhibition has previously been shown to improve myeloma bone disease^28,29^. In contrast to BMPR1a-FC, we found an activin ligand trap (ACVR2a-FC) to slightly increase osteoclastogenesis (Fig. 4f, g), demonstrating that BMP and activin inhibition have non-equivalent effects on myeloma bone disease in this model system.

### BMP blockade reduces osteoblast suppression in myeloma

**Fig. 5:**
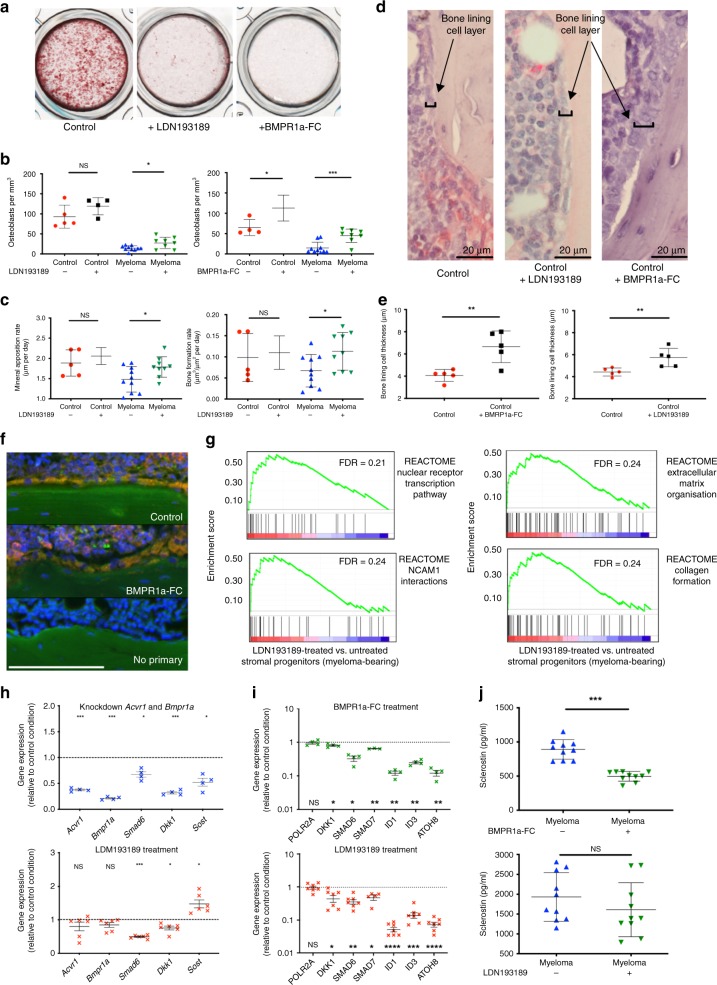
BMP inhibition promotes osteoblast differentiation in myeloma in vivo via down-regulation of Wnt inhibitors. **a** Mineralisation of murine pre-osteoblast cell line 2T3 in the presence of LDN193189 or BMPR1a-FC. **b**–**e** Histomorphometric analysis of haematoxylin- and TRAP-stained femur sections from healthy and myeloma-bearing mice: *n* = 5 per control group, 10 per myeloma group, total *n* = 30. Missing data points: see Methods. **b** Bone-lining cell density (N.Ob/T.Ar, per mm^3^) in mice treated with vehicle vs. LDN193189 and vehicle vs. BMPR1a-FC. **c** Mineral apposition rate (μm per day) and bone formation rate (μm^3^/μm^2^ per day) in healthy and myeloma-bearing mice treated with vehicle vs. LDN193189. **d** Images of bone-lining cell layer in healthy mice treated with vehicle, LDN193189 or BMPR1a-FC. **e** Bone-lining cell layer thickness (μm) in healthy mice treated with vehicle vs. BMPR1a-FC and vehicle vs. LDN193189. **f** Images of bone-lining cell layer in healthy mice treated with vehicle vs. BMPR1a-Fc, stained for osterix and vitamin D receptor (VDR). Blue = Hoescht, green = osterix, red = VDR, yellow/orange = expression of osterix and VDR. Scale bar = 100 μm. **g** Gene sets enriched (FDR <0.25, *n* > 25 genes) on GSEA of stromal progenitors from LDN193189 vs. vehicle-treated myeloma-bearing mice. **h** Effect of siRNA double knockdown of *Acvr1* and *Bmpr1a* (rat osteosarcoma line UMR-106.01) on *Acvr1*, *Bmpr1a*, *Smad6*, *Dkk1* and *Sost* (sclerostin) gene expression (*n* = 4 replicates). Effect of incubation with LDN193189 (100 nM, 24 h) on expression of same genes was performed for comparison (*n* = 6 replicates). **i** Expression of *Polr2a* (control) and BMP-response genes *Smad7*, *Sma6*, *Id1*, *Id3*, *Atoh8*, and *Dkk1* by myeloma patients’ bone marrow stromal cells after 24 h incubation with LDN193189 (100 nM) (*n* = 6 samples) or BMPR1a-FC (10 μg/ml) (*n* = 4 samples). *Sost* not detectable. **j** Bone marrow plasma sclerostin level in vehicle vs. BMPR1a-FC-treated and vehicle vs. LDN193189-treated myeloma-bearing mice. *P < 0.05, **p < 0.01, ***p < 0.001, NS, not significant. **b**–**e**, **j** *t* Test, **h** one-way ANOVA with Dunnett’s multiple comparison test and **i** paired ratio *t* test. Data represent mean ± SD

Myeloma bone disease results from both elevated osteoclast number and activity, and osteoblastic suppression. 2T3 preosteoblasts express endogenous BMP2 and undergo osteoblastic differentiation^30^. LDN and BMPR1a-FC were found to inhibit mineralisation of 2T3 cells in osteogenic media (Fig. 5a). However in vivo, histomorphometric analysis demonstrated significantly more BLCs following treatment of myeloma-bearing mice with LDN or BMPR1a-FC (Fig. 5b), although levels did not reach those of control non-tumour-bearing animals. Myeloma-bearing mice were found to increase mineral apposition and bone formation rates following LDN treatment (Fig. 5c). Blinded histological examination revealed that treatment with BMPR1a-FC, and to a lesser degree LDN, resulted in a thicker BLC layer (Fig. 5d–f), a morphological characteristic related to the conversion of quiescent BLCs to active osteoblasts and recently associated with inhibition of sclerostin^5^. BLCs were found to express both osterix and vitamin D receptor, supporting osteoblast development (Fig. 5f).

To investigate the mechanism by which BMP inhibition reduces osteoblast suppression in myeloma bone disease in vivo, we performed RNA-Seq analysis of stromal progenitors and osteoprogenitors isolated from myeloma-bearing mice treated with LDN or control. GSEA was performed to search for enriched gene sets in the Molecular Signatures Database curated gene set collection (MSigDB C2 collection, *n* = 3334 gene sets)^20^, and to investigate molecular associations of the increased bone mass observed. No enrichment of cell cycle-related gene sets was found, suggesting that BMP inhibition does not directly influence cell cycle (Supplementary Table 3). Four gene sets, all from the Reactome Database^24^, met the enrichment criteria (Fig. 5g, Supplementary Data 2): ‘Nuclear Receptor Transcription’, in which the most highly enriched gene was *Vdr*, vitamin D receptor (in top 20 most upregulated genes by LDN in stromal progenitor transcriptome, FDR 0.005); ‘Collagen formation’, ‘Extracellular matrix organisation’ and ‘NCAM1 interactions’. The enrichment of the latter three gene sets by LDN in the stromal progenitor transcriptome was driven by overlapping content of high numbers of collagen genes associated with matrix formation and cell–matrix adhesion, suggesting differentiation towards an osteoblast phenotype (Supplementary Data 2). In separate studies, stromal progenitors were isolated from myeloma-bearing mice following treatment with BMPR1a-Fc or control. A significant increase in alkaline phosphatase activity and *Runx2* expression, but not osterix or osteocalcin, was detected in response to in vivo treatment with BMPR1a-Fc, supporting a role for BMP inhibition in the early stages of osteoblast differentiation (Supplementary Fig. 17).

Reciprocal control between Wnt and BMP signalling is well described in normal bone development^31^. Impaired osteoblast differentiation in myeloma has previously been attributed to excessive Wnt inhibition, with particular implication of Wnt inhibitors Dkk1 and sclerostin. They have also previously been identified as downstream targets of BMP signalling^32,33^. In support of this, we found that single or double knockdown of *Bmpr1a* and *Acvr1* in the osteogenic UMR-106.01 cell line resulted in decreased expression of both *Dkk1* and *Sost* (sclerostin) gene expression and a reduction in secreted sclerostin (Fig. 5h; Supplementary Fig. 18). Double knockdown of *Bmpr1a* and *Acvr1* also decreased the BMP target gene *Smad6* (Fig. 5h). In contrast, LDN treatment only caused a decrease in *Dkk1*, but not *Sost* mRNA expression (Fig. 5h). To investigate whether BMP inhibition had a direct effect on Wnt inhibitor expression in myeloma patients, we cultured human bone marrow stromal cells isolated from patients with myeloma or monoclonal gammopathy of unknown significance (MGUS) (characteristics in Supplementary Table 4), and treated them with either LDN or BMPR1a-FC. Expression of *DKK1* and a range of BMP target genes was reduced by both treatments (expression of osteocyte-specific *SOST* was not detected in these stromal cells) (Fig. 5i). Additionally, sclerostin concentrations within the bone marrow plasma of myeloma-bearing mice were significantly reduced by BMPR1a-FC in both 5TGM1 and JJN-3-myeloma-bearing mice, but not LDN193189 (Fig. 5j; Supplementary Fig. 19). Taken together, these results suggest that reduction in expression of local Wnt inhibitors may be a mechanism by which BMP inhibition improves bone formation in myeloma-bearing mice.

## Discussion

This study found that transcriptomic profiling of the bone microenvironment implicated BMP signalling in myeloma pathogenesis, and provides preclinical evidence for inhibition of BMP signalling in the treatment of myeloma bone disease. Specific transcriptional changes occur in the BLCs of myeloma-bearing mice, particularly in stromal progenitors, where we found BMP pathway genes to be upregulated. BMP pathway inhibition using LDN193189 (LDN, an inhibitor of all BMP type 1 receptors, but with highest affinity for ACVR1)^23,34^ and BMPR1a-FC (a ligand trap with the FC domain of IgG1 attached to BMPR1a, leading to ligand removal) increased bone volume, decreased osteoclastogenesis and increased osteoblast differentiation in myeloma-bearing mice. A blocking antibody to BMP6 did not have these effects on bone homeostasis, but did improve hepcidin-mediated anaemia.

Previously published findings relating to BMP signalling in myeloma are limited to observations that BMPs are toxic to myeloma cells in vitro^11,13,35^, that BMPs may increase hepcidin levels and contribute to anaemia^36^ and that certain ligands and receptors may be increased in myeloma marrow samples, including BMP6^27,37^. It has been proposed that BMP ligands could have a therapeutic role in myeloma due to their myeloma-toxic and pro-osteogenic effects^13,38^. However, we found BMP-induced myeloma cell death to be abrogated by stromal contact, and in vivo no increase in overall tumour burden with BMP inhibition. Moreover, our analyses revealed no change in gene sets associated with tumour growth and survival in myeloma cells following in vivo BMP inhibition. Indeed, in the JJN-3 model of myeloma, we found a small reduction in overall tumour burden, possibly reflecting an indirect anti-tumour effect in response to reducing bone disease. However, we cannot definitively exclude the possibility that there could be long-term effects of BMP inhibition to worsen tumour burden.

Increased bone mass has been previously reported in inducible BMPR1a-knockout mice, with disruption of BMPR1a in osteoblasts, osteoclasts or osteocytes, and with type 1 BMP receptor blockade using BMPR1a-FC in an osteoporosis model^26,32,39–41^. In contrast, BMPR1b-null mice are reported to be osteopenic, with no change in osteoclastic bone resorption and attributed to compromised osteoblast differentiation^42^. Our findings expand these observations to the severe and complex bone disease found in myeloma. The increased bone mass was accompanied by reduced osteoclast numbers and serum TRAP-5b levels. Aberrant osteoclastic activity in myeloma bone disease is associated with an increase in the RANKL to OPG ratio; we found that this was not corrected in osteoblasts following LDN treatment in vivo. While we cannot exclude the possibility that BMP blockade may also indirectly reduce myeloma bone disease via direct effects on tumour cells, the direct inhibition of osteoclastogenesis in vitro by LDN and BMPR1a-FC suggests the effect is not mediated by tumour or other components of the microenvironment, consistent with a previous description of direct osteoclastogenic activity of BMPs^43^. In marked contrast, in the same experimental conditions, inhibition of the related activin pathway using an activin ligand trap increased osteoclast number. This highlights that the mechanism of bone mass increase seen on BMP blockade is not biologically interchangeable with that seen on activin blockade.

The exact stage(s) of osteogenic differentiation blocked by myeloma are unknown. We sorted BLCs from mice with early myeloma infiltration to ensure they could be collected; in later disease this population becomes extremely scarce. Using the methodology described by Nakamura et al.,^15^ our sorted population of Sca1+ALCAM− cells is enriched for stromal progenitor cells, as demonstrated by their ability to differentiate into adipocytes and osteoblasts and their gene expression profile (Fig. 1b; Supplementary Fig. S3). In contrast, our sorted population of ALCAM+Sca1− cells are enriched for osteoprogenitor cells, as demonstrated by their ability to differentiate into osteoblasts, but not adipocytes, and their gene expression profile. In addition to ‘bone loss’ gene set enrichment in stromal progenitor cells, we also observed ‘bone formation’ gene set enrichment. In early stages of myeloma bone disease, osteoblasts are reportedly increased, in an attempt to maintain normal coupling between osteoclasts and osteoblasts in the face of increased osteoclastic activity^44^. Therefore, it is not surprising that mixed features of blocked and activated osteoblast differentiation were seen.

Successful myeloma bone disease therapeutics may achieve higher bone mass either by reducing bone resorption, akin to current clinical therapies that target osteoclasts alone (bisphosphonates and denosumab), or by increasing bone anabolism through modification of myeloma-induced osteoblast differentiation block. Although there is hope that anti-sclerostin antibodies may offer this type of bone repair potential, there is no such therapy currently available for clinical use. Our in vitro experiments indicate that BMP inhibition does not directly stimulate osteoblast precursors to promote bone formation; indeed, as expected, in an isolated osteoblast culture system where endogenous BMP2 supports osteoblast differentiation^30^, both LDN193189 and BMPR1a-Fc inhibit mineralisation. This suggests that our observed in vivo bone anabolic effect is environmentally mediated. We hypothesised that BMP inhibition might recapitulate the anabolic effects of anti-sclerostin antibodies in myeloma bone disease in vivo, since: (a) osteocyte-secreted sclerostin is a major mediator of the osteogenesis block in myeloma;^45^ (b) anti-sclerostin antibodies ‘activate’ quiescent BLCs to become thicker, active and produce bone matrix in vivo^5^ and (c) both *Acvr1* and *Bmpr1a* knockout in vivo decreased *Sost* and *Dkk1* transcription^32,39^.

BMPR1a inhibition has been previously noted to improve collagen structure and strength^46^, and our data showing upregulation of collagen genes in stromal progenitors from LDN-treated mice supports this. The additionally enriched ‘Nuclear Receptor Transcription’ gene set represents a group of hormone receptors and their associated nuclear-binding apparatus, including the vitamin D receptor. The *Vdr* gene was highly enriched in stromal progenitors from LDN-treated myeloma-bearing mice. *Vdr* is a marker of developing osteoblasts, reported to have a role in ensuring osteogenic rather than adipogenic differentiation, and in osteoblast to osteocyte transition, controlling downstream bone matrix formation gene networks^47–49^. Dual staining for VDR and osterix was present in the BLC layer with greater thickness following BMP inhibition. Our GSEA data support the hypothesis that BMP inhibition alters the transcriptome of stromal progenitors in favour of osteoblastic differentiation. Knockdown of *Acvr1* and *Bmpr1a* together or separately in an osteocyte cell line, in mimicry of the inhibitory effects of these drugs, suppressed the production of Dkk1 and sclerostin. LDN and BMPR1a-FC could inhibit Dkk1 expression in bone marrow mesenchymal stem cells (BMSCs) from myeloma patients, but only BMPR1a-FC reduced sclerostin levels in the bone marrow plasma of myeloma-bearing mice, which may explain the stronger anabolic effect of BMPR1a-FC.

Interestingly, we detected an increase in the small proportion of myeloma cells adherent to the BLC layer on treatment with LDN. Since a proposed purpose of bone-lining niche disruption by myeloma cells is deregulation of the dormant tumour fraction in favour of proliferation^50^, it is intriguing to speculate that the increase in BLCs in response to BMP blockade may facilitate an increase in the dormant myeloma population, potentially desirable during MGUS or remission states.

We were also interested in the overlap of effect of BMP pathway inhibition on bone disease and anaemia: first, both are important complications in patients with myeloma; and second, other ligand traps of the TGFβ superfamily reverse anaemia^51^, or in the case of ACVR2A-FC, improve anaemia and myeloma bone disease^52^. However, the haemoglobin-increasing mechanisms of these drugs are not reported to be hepcidin-mediated, whereas antagonism of type 1 BMP receptor signalling reduces hepcidin and inflammatory anaemia^53^. Anaemia in the 5TGM1 model of myeloma is variable and unpredictable, likely reflecting the speed of disease development. Although LDN and BMPR1a-FC reduced hepcidin expression in vitro, and have reduced anaemia in inflammatory anaemia models^53^, neither approach increased haemoglobin in 5TGM1 myeloma-bearing mice, but BMPR1a-Fc did increase haemoglobin in JJN-3 myeloma-bearing mice. Anti-BMP6, which is highly effective at suppressing hepcidin, increased iron availability and improved haemoglobin in 5TGM1 myeloma-bearing mice, suggesting a specific role for BMP6 in the anaemia associated with myeloma.

**Fig. 6:**
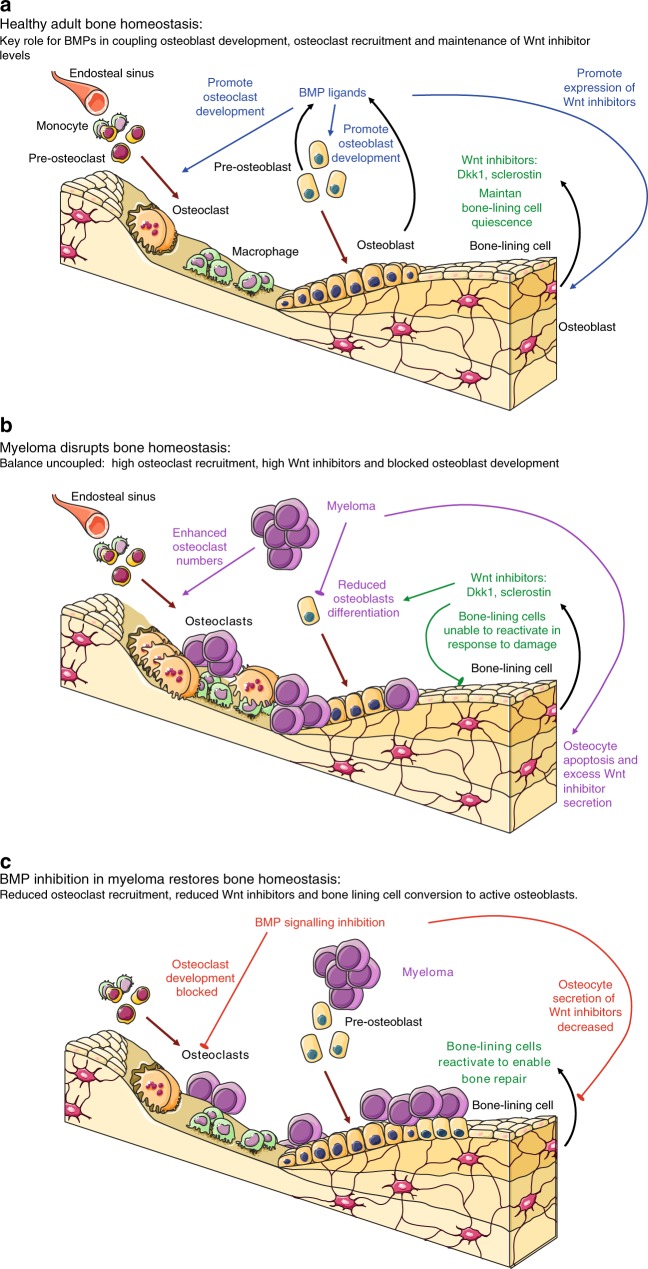
Mechanistic model of the involvement of BMP signalling inhibition in the restoration of bone mass in multiple myeloma. **a** BMP signalling in adult bone homeostasis couples osteoblast and osteoclast development. By controlling expression of Wnt inhibitors, BMP signalling also prevents excess matrix deposition. Wnt inhibitors are negative regulators of osteoblast development and bone-lining cell reactivation. **b** Myeloma overrides the balance between osteoclast and osteoblast leading to excess bone resorption without repair. High Wnt inhibitor levels, induced by myeloma-induced osteocyte toxicity and apoptosis, are critical to this uncoupling by preventing osteoblast development and bone-lining cell activation. **c** Inhibition of BMP signalling reverses the excess Wnt inhibitor levels and osteoclast recruitment, thereby allowing bone-lining cells to reactivate to matrix-secreting osteoblasts to restore damage. Figure produced using Servier Medical Art (http://www.servier.com, licensed under a creative commons attribution 3.0 unported licence)

In summary, in bone-lining stromal progenitors, we found BMP pathway members, particularly *Bmpr1b*, to be enriched in myeloma-bearing mice. BMP inhibition leads to increased bone mass, through a direct anti-osteoclast effect and a reduction in Wnt inhibitor production, particularly sclerostin by osteocytes, rather than through a direct effect on stromal progenitors or preosteoblasts. The bone mass-enhancing effect of the BMP blockade described here occurs in control and diseased mice, so is a generic effect on both diseased and healthy bones. However, by causing a reduction in pathologically high sclerostin levels and so promoting osteoblast differentiation, whilst also inhibiting osteoclast development, pharmacological BMP inhibition has the potential to overcome the uncoupling of bone homeostasis that drives myeloma bone disease (Fig. 6).

## Methods

### In vivo studies

The KaLwRiJ 5T murine myeloma model was selected for these investigations due to its faithful recapitulation of the lytic lesions of human myeloma, combined with immunocompetence, which is critical to accurately model niche–tumour interactions. We have complied with all relevant ethical regulations for animal testing and research. Experiments were approved by the University of Oxford Animal Welfare Ethical Review Body (Home Office Project License 30/2996). C57Bl/KaLwRijHsd mice (Harlan Netherlands BV, The Netherlands) or NOD/SCID-GAMMA (NSG: NOD.Cg-Prkdcscid Il2rgtm1Wjl/SzJ, Charles River laboratories, UK) were used. Taking into consideration ‘3Rs’ guidance in use of animals for scientific research, standard sample size for pharmacological intervention studies was *n* = 30 mice, 5 per control group, 10 per myeloma-bearing group (power analysis indicated that *n* = 10 mice per group would be required to detect a significant change in tumour burden). In the case of LDN intervention, for microCT purposes only, this was repeated once to total *n* = 60. In the anti-BMP6 intervention study, there were only myeloma-bearing groups. In separate studies, the JJN-3 murine myeloma model was used, with sample size of *n* = 26, 5 per control group and 8 per myeloma-bearing group^54^. Within each study, mice were age-, sex- and weight-matched between treatments. All assessments were performed by blinded assessors.

Animals were housed in individually ventilated cages in the Department of Biomedical Services, University of Oxford, with access to normal chow and water ad libitum. Eight- to 10-week-old C57Bl/KaLwRij mice were inoculated intravenously (IV) with phosphate-buffered saline (PBS) vehicle or 1 × 10^6^ 5TGM1-GFP cells (niche-sorting experiment) or 2 × 10^6^ 5TGM1-GFP (LDN193189, BMPR1a-FC and anti-BMP6 experiments). Seven- to eight-week-old NSG mice were inoculated IV with PBS vehicle or 1 × 10^6^ JJN-3 cells. Experiments for bone-lining niche sorting were ended prospectively on day 23. All other experiments were ended on the day the first mouse exhibited signs of hindlimb weakness, typically days 21–25. All drugs were injected from day 5 post tumour inoculation until cull. LDN193189 (Sigma) or vehicle (20% (2-hydroxypropyl)-β-cyclodextrin) was injected intraperitoneally (IP) daily at 3 mg/kg. BMPR1a-FC (Acceleron Pharma) was injected subcutaneously (SC) twice per week at 10 mg/kg. Anti-BMP6 (Kymab Ltd., fully cross-reactive with mouse Bmp6) was injected IP three times per week at 10 mg/kg. In one experimental replicate using LDN193189 (*n* = 30), 0.4 mg calcein was injected IP twice in the week preceding cull to enable measurement of bone formation rate.

### Bone-lining niche cell isolation

Bilateral femora and tibiae were cleaned, crushed, repeatedly washed to remove all marrow and then minced finely with scissors. Fragments were incubated in sterile 37 °C 3 mg/ml type 1 collagenase (Worthington) in PBS (5% foetal calf serum (FCS), 200 U/ml DNAse1) on rotation for 30 min to allow matrix digestion, after which the released cell suspension was filtered, washed and stored on ice in PBS (5% FCS 2 mM EDTA). Bone fragments were subjected to a second 30-min incubation in fresh collagenase solution. Cells released from the second incubation were added to those from the first, and all cells treated with FC block were then stained with antibody cocktail (antibodies detailed in Supplementary Table S5)^15^. Using a BD FACSAria III Cell Sorter (BD Biosciences), 100 cells per sample were sorted into 4 µl lysis buffer (FACS plots are shown in Supplementary Fig. S1) and processed for RNA-Seq using the SmartSeq2 protocol^55^. Complementary DNA (cDNA) libraries were sequenced using Illumina HiSeq2000 (50 bp single-end reads, healthy vs. myeloma-bearing) or HiSeq4000 (75 bp paired-end reads, vehicle vs. LDN193189-treated myeloma-bearing) at the Wellcome Trust Centre for Human Genetics, University of Oxford. In separate experiments, BLCs were sorted and cultured in osteogenic or adipogenic conditions.

### RNA-Seq data analysis

Following quality control (QC) analysis (Supplementary Fig. 2) with the fastQC package (http://www.bioinformatics.babraham.ac.uk/projects/fastqc), reads generated from sequencing were aligned using STAR^56^ against the mouse genome assembly (Genome Reference Consortium GRCm38 (mm10) UCSC transcripts). For the paired-end data, the reads that were identified as PCR duplicates using Samtools^57^ were discarded. Post mapping quality control was assessed using RNA-SeQC^58^ (Supplementary Fig. 2). Gene expression levels were quantified as read counts using the featureCounts command^59^ from the Subread package^60^ (http://subread.sourceforge.net) with default parameters. Only genes with more than one count per million in at least four samples were kept for any downstream analysis. The read counts were used for the identification of global differential gene expression between specified populations using the voom-limma method^17–19^. RPKM (Reads Per Kilobase of transcript per Million mapped reads) values and the MDS plot were generated using the edgeR package^17^. Genes were considered differentially expressed between populations if they had an adjusted *p* value (FDR) <0.1 and abs(log FC) >1. Heatmaps were generated using the package pheatmap (https://CRAN.R-project.org/package = pheatmap).

RPKM values were analysed using GSEA to identify significantly enriched pathways^20^. For pre-designed bone homeostasis and BMP pathway gene sets, FDR of <0.05 was used to identify significant enrichment (Fig. 1d). For MSigDB C2 collection gene sets (http://software.broadinstitute.org/gsea/msigdb/collections.jsp#C2) (*n* = 3334), due to the high number concurrent gene sets assessed, by convention an FDR of <25% was adopted as a criterion for enrichment, in addition to gene set size >25.

### Mouse serum, bone marrow, bone and liver analysis

Right tibiae were formalin-fixed and microCT (Skyscan 1172 X-ray microtomograph; Bruker MicroCT, Kontich, Belgium) performed at 37 kV/228 μA, with an isometric resolution of 9.94 μm per pixel using a 0.5-mm aluminium filter. Reconstruction of the original scan data was performed using NRecon. The same threshold setting for bone tissue was used for all samples. On the three-dimensional reconstructed image, osteolytic lesions on the curved medial tibial surface that completely penetrated the cortical bone and were >100 μm in diameter were counted. Formalin-fixed, decalcified, paraffin-embedded right femorae were sectioned and stained with haematoxylin and eosin and for TRAP activity^61^. Histomorphometric analyses were performed using the OsteoMeasure software (OsteoMetrics, Decatur, GA, USA). Bone formation rate using calcein incorporation was performed on non-decalcified methylmethacrylate-embedded vertebral sections, also using the OsteoMeasure software. For immunohistochemistry, 4-μm slices of formalin-fixed, paraffin-embedded decalcified tibia were rehydrated and epitopes revealed by proteinase K digestion (Millipore Cat#21627, 20 μg/ml for 15 min). Primary antibody was applied overnight at 4 °C: 1:200 anti-VDR (LifeTech MA1-710) and 1:200 anti-OSX Abcam ab22552. Secondary antibody was applied for 1 h at room temperature: 1:200 anti-rat-AF488 (LifeTech A-11006) and 1:200 anti-rabbit-AF568 (LifeTech A-11011), with 2 μg/ml Hoechst 33342. Slides were coverslipped and sealed with Diamond Antifade (Thermo), and then viewed on fluorescent microscope.

Blood was taken by cardiac puncture immediately after culling mice. One hundred and fifty microliters stored in BD Microtainer EDTA tubes (Beckton Dickinson) was used to measure plasma haemoglobin concentration within 6 h (Sysmex KX-21N-automated haematology analyser). Serum was prepared by the centrifugation of clotted blood at >6000 g for 3–5 min in BD Microtainer SST tubes (Beckton Dickinson); serum aliquots were frozen immediately at −80 °C. Serum IgG2b concentration was quantified by enzyme-linked immunosorbent assay (ELISA) (Mouse IgG2b ELISA Quantitation Set, Bethyl Laboratories). Serum kappa free light chain was quantified using Seralite®-FLC ELISA (Abingdon Health). Serum TRAcP5b enzyme activity was quantified using MouseTRAP^TM^ ELISA (Immunodiagnostic Systems). Serum hepcidin concentrations were quantified using the Hepcidin-Murine Compete ELISA Kit (Intrinsic Lifesciences). Serum iron was quantified using the MULTIGENT Iron Kit on the Abbott Architect c16000 automated analyser (Abbott Laboratories). Murine liver explants were stored in RNAlater (Ambion) and homogenised by TissueRuptor (Qiagen), before RNA extraction and quantitative reverse transcription PCR (RT-qPCR). The left tibia and femur were crushed and bone marrow flushed out with exactly 1.5 ml PBS. This PBS was frozen immediately after centrifugation at −80 °C as bone marrow plasma. Bone marrow plasma sclerostin level was measured by Magnetic Luminex Screening Assay (R&D Systems). Bone marrow cell suspensions were filtered (70-µm filter) and analysed for percentage GFP fluorescence or, in the case of the JJN-3 model, the proportion of CD45−. GFP+, human BCMA+ JJN-3 myeloma cells, using a Cyan-ADP Flow cytometer (Beckman Coulter).

### Donated human tissue studies

Peripheral blood mononuclear cells were isolated from pooled human leucocyte cones purchased from NHS Blood and Transplant, and osteoclasts differentiated, as below^62^. Experimental treatments (LDN 100 nM, BMPR1a-FC or ACVR2a-FC 10 μg/ml) were added to media from day after seeding until mature osteoclast formation in control wells. Use of pooled human leucocyte cones for this work was approved by the Oxford Clinical Research Ethics Committee (C01.070). Patients with or under investigation for myeloma consented to the use of bone marrow aspirate samples for research purposes. This work was approved by Oxford Clinical Research Ethics Committee (09/H0606/5 project 13/A238). Mononuclear cells were isolated from fresh bone marrow aspirates using Histopaque (Sigma), and seeded onto tissue culture plates in α-minimum essential medium (α-MEM) culture media containing 10% FCS, l-glutamine (2 mM) and antibiotics. Adherent cells (BMSCs) were grown to confluence, reseeded and treated for 24 h with LDN 100 nM or BMPR1a-FC 10 μg/ml, before RNA extraction and RT-qPCR.

### Apoptosis in vivo

Paraffin-embedded sections of murine femur where deparaffinised and hydrated through graded alcohols to water and then subsequently immersed in citric acid buffer and steamed for 20 min. Sections were then treated with proteinase K (20 µg/ml) for 15 min and then permeabilised in 0.1% Tween for 5 min. ApopTag® Red In situ Apoptosis Detection Kit (S7165 Millipore) protocol was then followed as per the manufacturer’s instructions. Before anti-digoxigenin-rhodamine was added the sections were incubated overnight with 1:500 anti-GFP (Abcam ab13970). Secondary anti-digoxigenin-rhodamine and 1:200 anti-chicken AF488 (LifeTech A11039) were then added for 1 h at room temperature (RT). Sections were mounted using Diamond Antifade (Thermo). Staining was assessed using confocal microscopy, 3 fields of view for each section were taken at ×20 magnification and analysed using image J.

### Cell lines

Huh7 cells (an immortalised human hepatoma cell line^63^) were a gift of Prof. Persephone Borrow, University of Oxford. UMR-106 cell line (ECACC, 90111314) was a gift of Dr. Philippa Hulley, University of Oxford. JJN-3 (DSMZ, ACC 541) and MM1-S (ATCC CRL-2974) cell lines were a gift of Prof. Udo Oppermann, University of Oxford. JJN-3 myeloma cells (DSMZ, ACC 541) used for in vivo studies were a gift of Dr. Shelly Lawson, University of Sheffield. 2T3 mouse preosteoblasts^30^ were a kind gift from Dr. Steve Harris, University of Texas Health Science Center at San Antonio and HS5 human stromal cell line were obtained from ATCC (CRL-11882). 5TGM1 and 5TGM1-GFP murine myeloma cells^64^ were a kind gift from Prof. Gregory Mundy, University of Texas Health Science Center at San Antonio. All cell lines tested negative for mycoplasma.

### In vitro cell line studies

HuH7, UMR-106, 2T3 and HS5 cell lines were maintained in Dulbecco’s minimum essential medium supplemented with 10% FCS, 1% glutamine, and 1% penicillin/ streptomycin.

JJN-3, MM1-S, 5TGM1 and 5TGM1-GFP cells were maintained in RPMI-1640 supplemented with 10% FCS, 1% non-essential amino acids, 1% sodium pyruvate, 1% glutamine, and 1% penicillin/ streptomycin. AlamarBlue® cell viability assays were performed in RPMI supplemented as above, but with 2% FCS. Human BMP6 (R&D Systems) and/or LDN193189 (Sigma) were added at the time of plating. After 72 h incubation, 10% AlamarBlue® (10 μl) was added and the fluorescence was measured after 3 h. For fluorescence expressing cell contact experiment, HS5 cells were seeded for culture and allowed to adhere for 4 h. Media were replaced with/without additional experimental treatments and/or 5TGM1-GFP cells. Plate fluorescence was read at 72 h.

For 2T3 mineralisation assay, 48 h after seeding cells were treated every 3 days with osteogenic media comprising 2% β-glycerophosphate, 150 μM l-ascorbic acid and L-thyroxine 50 ng/ml and experimental treatments as indicated. Ossification was assessed by Alizarin Red staining.

For small interfering RNA (siRNA)-mediated gene knockdown experiments, rat UMR-106 cells were plated at 50% confluency; after 8 h, they were transfected with siRNA targeting rat Acvr1 and rat Bmpr1a (siGenome Smartpool, Dharmacon) using Lipofectamine RNAiMAX (Thermo Fisher), Opti-MEM (Thermo Fisher) and antibiotic-free media. The total amount of siRNA added was 100 nM. Cells were harvested after a further 48 h. LDN193189 (100nM) was added to equivalent wells 24 h before harvest. RNA extraction and RT-qPCR were performed as above.

### Osteoblast differentiation

Primary Sca1+ALCAM− and ALCAM+Sca1− cells were expanded in complete Mesencult medium (mouse, SCT). Cells were then re-plated at 1 × 10^3^/well (96-well plate), and once 60% confluent, media were replaced with Lonza Osteogenic BulletKit complete medium or control medium. At day +14, alkaline phosphatase activity was assessed (Sigma-Aldrich Alkaline Phosphatase Kit) using fast blue RR salt staining and ossification was assessed using Alizarin Red.

In separate experiments, cells were grown in α-MEM containing 20% FBS, 1% glutamine and 1% penicillin/streptomycin for 10 days. Cells were then treated with osteogenic media containing 10% FBS, 10 mM β-glycerophosphate, 10 nM dexamethasone and 150 μM l-ascorbic acid phosphate. Media were changed every 3 days. Alkaline phosphatase activity was assessed (Sigma-Aldrich Alkaline Phosphatase Kit, using fast violet B salt staining). Ossification was assessed using Alizarin Red.

### Adipocyte differentiation

Primary cells were expanded and re-plated as for osteoblast differentiation. Once 100% confluent, media were replaced with Lonza Adipogenic BulletKit complete induction medium, and adipogenesis cycles were performed as per the kit protocol. At day +21, adipogenesis was assessed using Oil Red O stain and BODIPY FL dye. Adiponectin levels were measured in conditioned media taken at days +3 and +21 (R&D Mouse Adiponectin Quantikine ELISA).

### Osteoclast differentiation

Positively selected CD14+ monocytes were seeded onto tissue culture plates in α-MEM culture media (without ribonucleosides/deoxyribonucleosides) containing 10% FCS, l-glutamine (2 mM), penicillin (50 IU/ml) and streptomycin sulfate (50 μg/ml). Cultures were supplemented with macrophage colony-stimulating factor (R&D Systems, 25 ng/ml), RANKL (gift Dr. J Dunford, 35 ng/ml) and experimental treatments either OPG (Peprotech, 500 ng/ml), LDN193189 (Sigma, 100 nM), human BMPR1a-FC (gift Dr. E. Martinez Hackert 10 µg/ml), or human ACVR2a-FC (gift Dr. E. Martinez Hackert 10 µg/ml), every 3–4 days, with mature osteoclasts being formed by day 9. Cells were fixed in 4% formalin and tartrate-resistant acid phosphatase (TRAP) was visualised using naphthol AS-BI phosphate as a substrate, with reaction of the product with Fast Violet B salt. TRAP-positive multi-nucleated cells containing ≥3 nuclei were considered osteoclasts.

### Quantitative real-time PCR

Gene expression in mouse liver tissue and in vitro assays as below was performed on RNA extracted using RNEasy Plus Kit (Qiagen) and reverse transcribed using High Capacity RNA-to-cDNA Kit (Applied Biosystems). RT-qPCR was performed on 7500Fast or QuantStudio7 instruments (Applied Biosystems) using inventoried TaqMan Gene Expression assays (Supplementary Table 6) and TaqMan Gene Expression Master Mix (Applied Biosystems). Changes in gene expression relative to endogenous controls (Hprt1 for mouse and rat, GAPDH for human) were calculated using the 2 − ΔCT method.

### Design of bone homeostasis gene sets

Gene set databases MSigDB, GO, BioCarta and KEGG were searched for gene sets relating to catabolic and/or anabolic aspects of bone turnover.

(A) Bone formation: The above databases were searched using terms ‘bone’, ‘ossification’, ‘mineralization’ and ‘osteoblast’. Members of all gene sets concerned with the positive regulation of bone formation or negative regulation of bone loss were screened, but individual genes within sets only included in the final list if the following criteria were met:

1. Genes were expressed in MSCs or their osteoblast lineage derivatives, in *Mus musculus* or *Rattus norvegicus*.
2. There was experimental evidence, ideally from animal knockout models, that bone formation was enhanced by (in any way and at any stage), or dependent on, expression of the gene in question, in the MSC lineage.

Genes were not included if these criteria were not met. Members of the following gene sets were included:

GO:0002076—osteoblast development

GO:0045669—positive regulation of osteoblast differentiation

GO:0002051—osteoblast fate commitment

GO:0033690—positive regulation of osteoblast proliferation

GO:0001503—ossification

GO:0045779—negative regulation of bone resorption

GO:0045671—negative regulation of osteoclast differentiation

GO:2001205—negative regulation of osteoclast development

GO:0030501—positive regulation of bone mineralisation

In addition, genes in the ‘bone remodelling’ gene set described below were added to this gene set if not already present, if they met ‘pro-bone formation’ inclusion criteria above. The final set comprised 198 members (Supplementary Data 1).

(B) Bone loss: This gene set was designed using an identical process to the ‘bone formation’ set, but reversing the criteria to include gene sets concerned with the negative regulation of bone formation or positive regulation of bone loss. Individual genes within sets were only included in the final list if the following criteria were met:

1. Genes were expressed in MSCs or their osteoblast lineage derivatives, in *Mus musculus* or *Rattus norvegicus*.
2. There was experimental evidence, ideally from animal knockout models, that bone loss was enhanced by (in any way and at any stage), or dependent on, expression of the gene in question, in the MSC lineage.

Genes were not included if these criteria were not met. Members of the following gene sets were included:

GO:0045668—negative regulation of osteoblast differentiation

GO:0033689—negative regulation of osteoblast proliferation

GO:0045780—positive regulation of bone resorption

GO:0030502—negative regulation of bone mineralisation

GO:0045672—positive regulation of osteoclast differentiation

GO:2001206—positive regulation of osteoclast development

GO:0090290—positive regulation of osteoclast proliferation

In addition, genes in the ‘bone remodelling’ gene set that were ‘pro-bone loss’, meeting inclusion criteria above, were added to this gene set if not already present. The final set comprised 154 members (Supplementary Data 1).

(C) Bone remodelling: The above gene set databases were searched using the term ‘bone remodel(l)ing’. Only genes expressed in MSCs and their derivatives, rather than those only expressed by osteoclasts or other inflammatory cells during bone remodelling, were included. The following gene sets were combined:

1. GO ‘Bone remodelling’ (GO:0046849), including all descendent GO term gene sets—limited to genes with evidence in *Mus musculus* or *Rattus norvegicus*.
2. MSigDB C2 gene set **ZAIDI**_OSTEOBLAST_TRANSCRIPTION_FACTORS, with additional genes added by consulting source material^65^.
3. Cancer Gene Anatomy Project (Biocarta) ‘bone remodelling’ pathway (https://cgap.nci.nih.gov/Pathways/BioCarta/h_ranklPathway).

The final set comprised 151 members (Supplementary Data 1).

**(**D) BMP signalling gene set: Forty four BMP pathway members (recognised ligands, inhibitors, receptors, intracellular signalling components and six-well-recognised targets) were included, if expressed by murine MSCs (Supplementary Data 1).

(E) Bone remodelling set with BMP pathway supplementation: Members of the BMP signalling pathway described above (but not already included) were added to bone remodelling gene set (Supplementary Data 1).

### Missing data values

Figures 2, 4, 5 and Supplementary Fig. 11 microCT and histomorphometric data have missing data values (noted in each legend) relative to total experimental *n*. This is due to broken/damaged bones at harvest, especially likely if severe bone disease is present. In Fig. 2l, two GFP percentage data values are missing from the myeloma + LDN cohort due to sample contamination.

### Statistical analyses

Analysis was performed with the GraphPad Prism Software (GraphPad Software Inc.). Where differences between treatment groups were experimentally hypothesised, means were compared using *t* tests. Multiple groupwise comparisons where differences between individual groups were not hypothesis driven were made by analysis of variance. Changes in osteoclast differentiation between paired-untreated/treated samples were assessed using paired *t* tests, and in patient BMSC gene expression experiments, paired ratio *t* tests with correction for false discovery rate by Benjamini and Hochberg method were used, due to baseline variation between patient samples. Significance was defined as *P* < 0.05. RNA-Seq statistical analysis is described above.

### Reporting summary

Further information on research design is available in the Nature Research Reporting Summary linked to this article.

## Supplementary information

Supplementary Information

Description of Additional Supplementary Files

Supplementary Data 1

Supplementary Data 2

Reporting Summary

Source Data

## Data Availability

RNA-Seq data have been deposited in NCBI’s Gene Expression Omnibus and are accessible through accession code GSE135786. Other data are available from the authors upon reasonable request. The source data underlying Figs. 2h, 2I and Supplementary Fig. 7 are provided as a Source Data file.

## References

[1] Kumar SK (2017). Multiple myeloma. Nat. Rev. Dis. Primers.

[2] Silbermann R, Roodman GD (2016). Current controversies in the management of myeloma bone disease. J. Cell. Physiol..

[3] Hauge EM, Qvesel D, Eriksen EF, Mosekilde L, Melsen F (2001). Cancellous bone remodeling occurs in specialized compartments lined by cells expressing osteoblastic markers. J. Bone Miner. Res..

[4] Kim SW (2012). Intermittent parathyroid hormone administration converts quiescent lining cells to active osteoblasts. J. Bone Miner. Res..

[5] Kim SW (2017). Sclerostin antibody administration converts bone lining cells into active osteoblasts. J. Bone Miner. Res..

[6] Reagan MR, Liaw L, Rosen CJ, Ghobrial IM (2015). Dynamic interplay between bone and multiple myeloma: emerging roles of the osteoblast. Bone.

[7] Eda H (2016). Regulation of sclerostin expression in multiple myeloma by Dkk-1: a potential therapeutic strategy for myeloma bone disease. J. Bone Miner. Res..

[8] McDonald MM (2017). Inhibiting the osteocyte-specific protein sclerostin increases bone mass and fracture resistance in multiple myeloma. Blood.

[9] Tian E (2003). The role of the Wnt-signaling antagonist DKK1 in the development of osteolytic lesions in multiple myeloma. N. Engl. J. Med..

[10] Manier S (2017). Genomic complexity of multiple myeloma and its clinical implications. Nat. Rev. Clin. Oncol..

[11] Kawamura C (2000). Bone morphogenetic protein-2 induces apoptosis in human myeloma cells with modulation of STAT3. Blood.

[12] Ro TB (2004). Bone morphogenetic protein-5, -6 and -7 inhibit growth and induce apoptosis in human myeloma cells. Oncogene.

[13] Holien T (2012). Bone morphogenetic proteins induce apoptosis in multiple myeloma cells by Smad-dependent repression of MYC. Leukemia.

[14] Radl J, Croese JW, Zurcher C, Van den Enden-Vieveen MH, de Leeuw AM (1988). Animal model of human disease. Multiple myeloma. Am. J. Pathol..

[15] Nakamura Y (2010). Isolation and characterization of endosteal niche cell populations that regulate hematopoietic stem cells. Blood.

[16] Hu X (2016). Identification of a common mesenchymal stromal progenitor for the adult haematopoietic niche. Nat. Commun..

[17] Robinson MD, McCarthy DJ, Smyth GK (2010). edgeR: a Bioconductor package for differential expression analysis of digital gene expression data. Bioinformatics.

[18] Law CW, Chen Y, Shi W, Smyth GK (2014). voom: precision weights unlock linear model analysis tools for RNA-seq read counts. Genome Biol..

[19] Ritchie ME (2015). limma powers differential expression analyses for RNA-sequencing and microarray studies. Nucleic Acids Res..

[20] Subramanian A (2005). Gene set enrichment analysis: a knowledge-based approach for interpreting genome-wide expression profiles. Proc. Natl. Acad. Sci. USA.

[21] Chen D (1998). Differential roles for bone morphogenetic protein (BMP) receptor type IB and IA in differentiation and specification of mesenchymal precursor cells to osteoblast and adipocyte lineages. J. Cell Biol..

[22] Sánchez-Duffhues G, Hiepen C, Knaus P, ten Dijke P (2015). Bone morphogenetic protein signaling in bone homeostasis. Bone.

[23] Mohedas AH (2013). Development of an ALK2-biased BMP type I receptor kinase inhibitor. ACS Chem. Biol..

[24] Fabregat A (2016). The Reactome pathway knowledgebase. Nucleic Acids Res..

[25] Yadin D, Knaus P, Mueller TD (2016). Structural insights into BMP receptors: specificity, activation and inhibition. Cytokine Growth Factor Rev..

[26] Baud’huin M (2012). A soluble bone morphogenetic protein type IA receptor increases bone mass and bone strength. Proc. Natl. Acad. Sci. USA.

[27] Seckinger A (2009). Bone morphogenic protein 6: a member of a novel class of prognostic factors expressed by normal and malignant plasma cells inhibiting proliferation and angiogenesis. Oncogene.

[28] Chantry AD (2010). Inhibiting activin-A signaling stimulates bone formation and prevents cancer induced bone destruction in vivo. J. Bone Miner. Res..

[29] Vallet S (2010). Activin A promotes multiple myeloma-induced osteolysis and is a promising target for myeloma bone disease. Proc. Natl. Acad. Sci. USA.

[30] Ghosh-Choudhury N (1996). Immortalized murine osteoblasts derived from BMP 2-T-antigen expressing transgenic mice. Endocrinology.

[31] Baron R, Kneissel M (2013). WNT signaling in bone homeostasis and disease: from human mutations to treatments. Nat. Med..

[32] Kamiya N, Kaartinen VM, Mishina Y (2011). Loss-of-function of ACVR1 in osteoblasts increases bone mass and activates canonical Wnt signaling through suppression of Wnt inhibitors SOST and DKK1. Biochem. Biophys. Res. Commun..

[33] Kamiya N (2010). Wnt inhibitors Dkk1 and Sost are downstream targets of BMP signaling through the type IA receptor (BMPRIA) in osteoblasts. J. Bone Miner. Res..

[34] Yu PB (2008). BMP type I receptor inhibition reduces heterotopic [corrected] ossification. Nat. Med..

[35] Hjertner O (2001). Bone morphogenetic protein-4 inhibits proliferation and induces apoptosis of multiple myeloma cells. Blood.

[36] Maes K (2010). In anemia of multiple myeloma, hepcidin is induced by increased bone morphogenetic protein 2. Blood.

[37] Grcevic D (2010). Bone morphogenetic proteins and receptors are over-expressed in bone-marrow cells of multiple myeloma patients and support myeloma cells by inducing ID genes. Leuk. Res..

[38] Seher A (2017). Utilizing BMP-2 muteins for treatment of multiple myeloma. PLoS ONE.

[39] Kamiya N (2008). Disruption of BMP signaling in osteoblasts through type IA receptor (BMPRIA) increases bone mass. J. Bone Miner. Res..

[40] Kamiya N (2016). Targeted disruption of BMP signaling through type IA receptor (BMPR1A) in osteocyte suppresses SOST and RANKL, leading to dramatic increase in bone mass, bone mineral density and mechanical strength. Bone.

[41] Okamoto M (2011). Conditional deletion of Bmpr1a in differentiated osteoclasts increases osteoblastic bone formation, increasing volume of remodeling bone in mice. J. Bone Miner. Res..

[42] Shi C (2016). Deletion of BMP receptor type IB decreased bone mass in association with compromised osteoblastic differentiation of bone marrow mesenchymal progenitors. Sci. Rep..

[43] Jensen ED (2010). Bone morphogenic protein 2 directly enhances differentiation of murine osteoclast precursors. J. Cell. Biochem..

[44] Bataille R (1991). Recruitment of new osteoblasts and osteoclasts is the earliest critical event in the pathogenesis of human multiple myeloma. J. Clin. Investig..

[45] Delgado-Calle J (2016). Bidirectional Notch signaling and osteocyte-derived factors in the bone marrow microenvironment promote tumor cell proliferation and bone destruction in multiple myeloma. Cancer Res..

[46] Zhang Y (2016). Loss of BMP signaling through BMPR1A in osteoblasts leads to greater collagen cross-link maturation and material-level mechanical properties in mouse femoral trabecular compartments. Bone.

[47] Wang Y, Zhu J, DeLuca HF (2014). Identification of the vitamin D receptor in osteoblasts and chondrocytes but not osteoclasts in mouse bone. J. Bone Miner. Res..

[48] Cianferotti L, Demay MB (2007). VDR-mediated inhibition of DKK1 and SFRP2 suppresses adipogenic differentiation of murine bone marrow stromal cells. J. Cell. Biochem..

[49] St John HC (2014). The osteoblast to osteocyte transition: epigenetic changes and response to the vitamin D3 hormone. Mol. Endocrinol..

[50] Lawson MA (2015). Osteoclasts control reactivation of dormant myeloma cells by remodelling the endosteal niche. Nat. Commun..

[51] Platzbecker U (2017). Luspatercept for the treatment of anaemia in patients with lower-risk myelodysplastic syndromes (PACE-MDS): a multicentre, open-label phase 2 dose-finding study with long-term extension study. Lancet Oncol..

[52] Abdulkadyrov KM (2014). Sotatercept in patients with osteolytic lesions of multiple myeloma. Br. J. Haematol..

[53] Steinbicker AU (2011). Inhibition of bone morphogenetic protein signaling attenuates anemia associated with inflammation. Blood.

[54] Lawson MA (2015). NOD/SCID-GAMMA mice are an ideal strain to assess the efficacy of therapeutic agents used in the treatment of myeloma bone disease. PLoS ONE.

[55] Picelli S (2014). Full-length RNA-seq from single cells using Smart-seq2. Nat. Protoc..

[56] Dobin A (2013). STAR: ultrafast universal RNA-seq aligner. Bioinformatics.

[57] Li H (2009). The Sequence Alignment/Map format and SAMtools. Bioinformatics.

[58] DeLuca DS (2012). RNA-SeQC: RNA-seq metrics for quality control and process optimization. Bioinformatics.

[59] Liao Y, Smyth GK, Shi W (2014). featureCounts: an efficient general purpose program for assigning sequence reads to genomic features. Bioinformatics.

[60] Liao Y, Smyth GK, Shi W (2013). The Subread aligner: fast, accurate and scalable read mapping by seed-and-vote. Nucleic Acids Res..

[61] Edwards CM (2008). Increasing Wnt signaling in the bone marrow microenvironment inhibits the development of myeloma bone disease and reduces tumor burden in bone in vivo. Blood.

[62] Knowles HJ, Cleton-Jansen AM, Korsching E, Athanasou NA (2010). Hypoxia-inducible factor regulates osteoclast-mediated bone resorption: role of angiopoietin-like 4. FASEB J..

[63] Nakabayashi H, Taketa K, Miyano K, Yamane T, Sato J (1982). Growth of human hepatoma cells lines with differentiated functions in chemically defined medium. Cancer Res..

[64] Dallas SL (1999). Ibandronate reduces osteolytic lesions but not tumour burden in a murine model of myeloma bone disease. Blood.

[65] Zaidi M (2007). Skeletal remodeling in health and disease. Nat. Med..

