# Supplementary material for: Transcriptomic profiling of the myeloma bone-lining niche reveals BMP signalling inhibition to improve bone disease
Source: Nat Commun. 2019 Oct 4;10:4533. doi: 10.1038/s41467-019-12296-1 (PMC6778199; doi:10.1038/s41467-019-12296-1)
Supplement: Supplementary file 1 — Supplementary Information [file 41467_2019_12296_MOESM1_ESM.pdf]

Transcriptomic profiling of the myeloma bone-lining niche reveals BMP signalling inhibition to improve bone disease

Gooding et al.

SUPPLEMENTARY INFORMATION

SUPPLEMENTARY FIGURES:

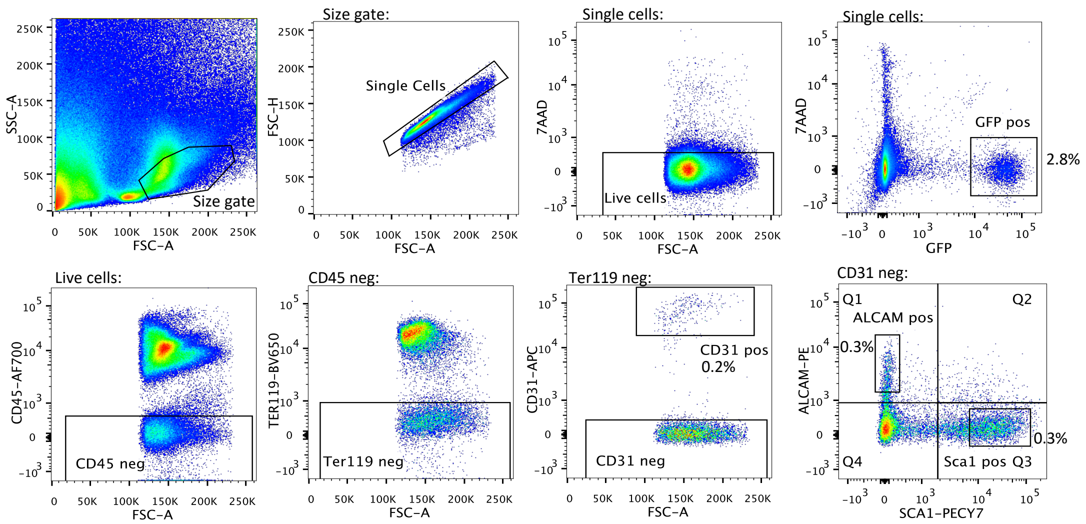

Supplementary Figure 1:

Full gating strategy used to FACS sort bone lining niche and 5TGM1-GFP myeloma cells

Plots representing the full gating strategy used to sort GFP-tagged 5TGM1 myeloma cells, CD45-/Ter119-/CD31+ endothelial cells, CD45-/Ter119-/CD31-/Sca1+ stromal progenitor cells and CD45-/Ter119-/CD31-/ALCAM+ osteoprogenitor cells. Percentages of sorted populations shown are all expressed as percentages of the live cell gate. The parent population of each plot is stated above the plot. For antibody conjugates used, see Supplementary Table S7.

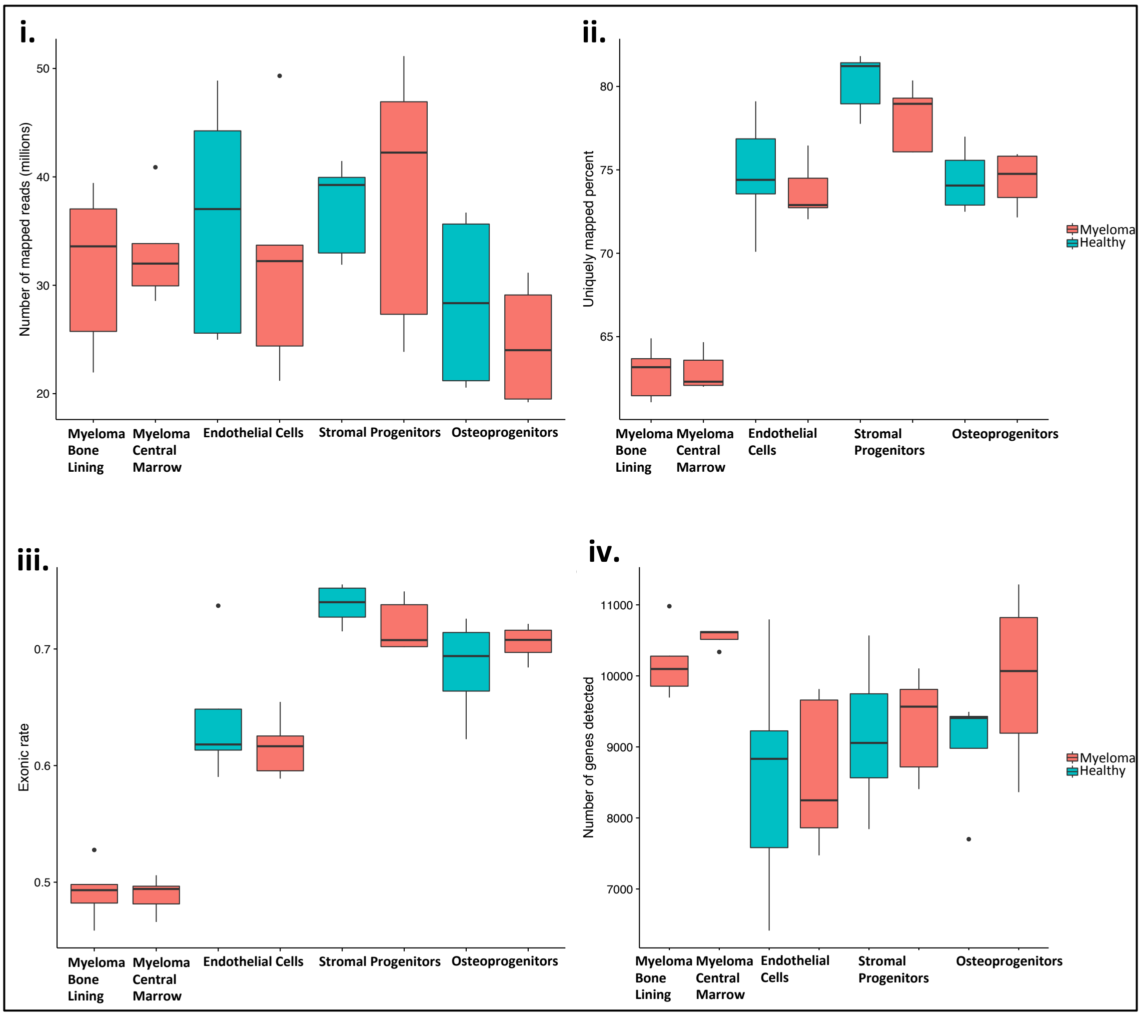
i.
Number of mapped reads (millions)
50
40
30
20
Myeloma Bone Lining
Myeloma Central Marrow
Endothelial Cells
Stromal Progenitors
Osteoprogenitors
ii.
Uniquely mapped percent
80
75
70
65
Myeloma Bone Lining
Myeloma Central Marrow
Endothelial Cells
Stromal Progenitors
Osteoprogenitors
Myeloma
Healthy
iii.
Exonic rate
0.7
0.6
0.5
Myeloma Bone Lining
Myeloma Central Marrow
Endothelial Cells
Stromal Progenitors
Osteoprogenitors
iv.
Number of genes detected
11000
10000
9000
8000
7000
Myeloma Bone Lining
Myeloma Central Marrow
Endothelial Cells
Stromal Progenitors
Osteoprogenitors
Myeloma
Healthy

A

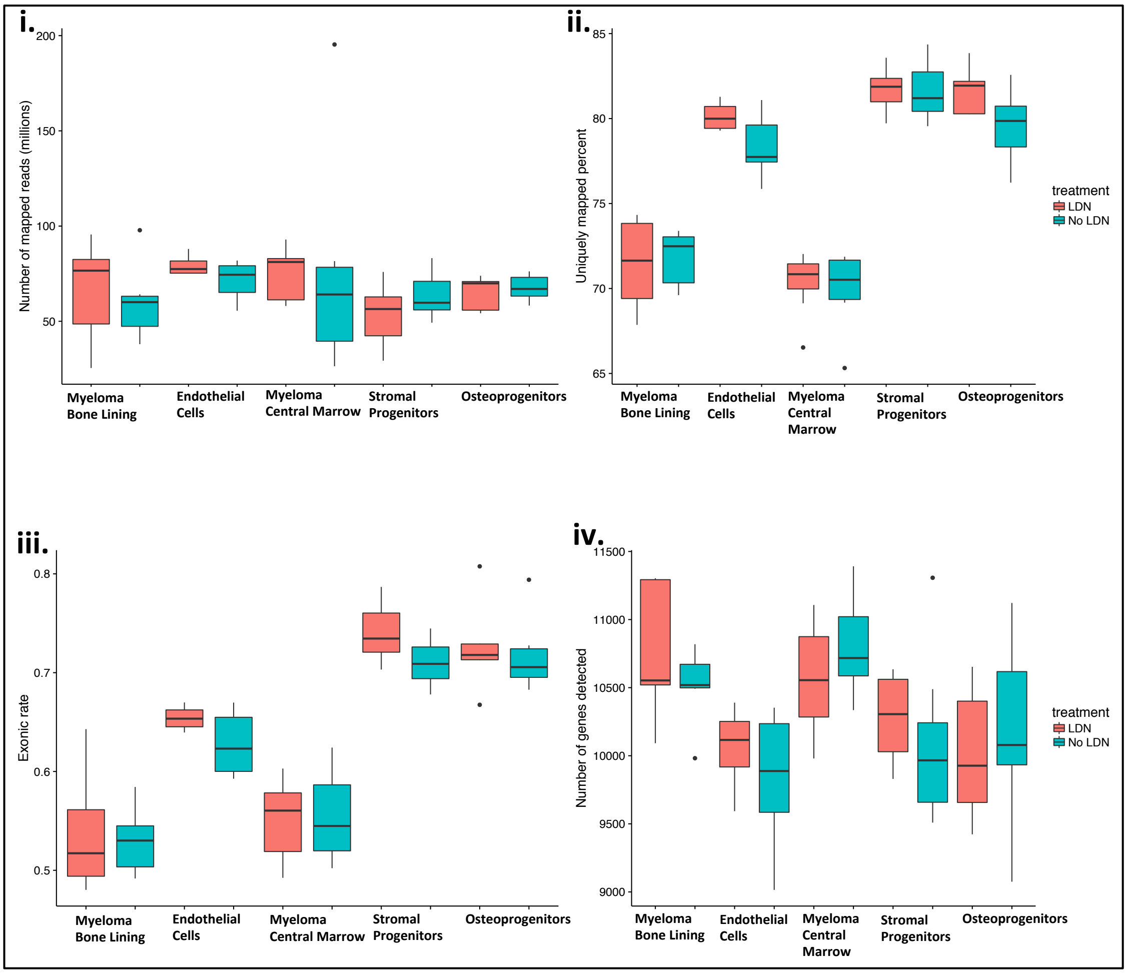

B

Supplementary Figure 2:

Quality control assessment of RNASeq mapping

(A) Healthy vs Myeloma-bearing mice: i) number and ii) percentage of reads mapping uniquely to the genome, iii) proportion of reads that map to annotated exonic regions and iv) number of genes that are expressed in each group of samples

(B) LDN-treated vs Non LDN-treated Myeloma-bearing mice: as above.

(A) and (B): The centre line is the median (50% quantile), box boundaries denote 25% quantile to the 75% quantile. Bottom whisker: smaller observation greater than or equal to lower box boundary - 1.5 * IQR (inter-quartile range). Top whisker: largest observation less than or equal to upper box boundary + 1.5 * IQR.

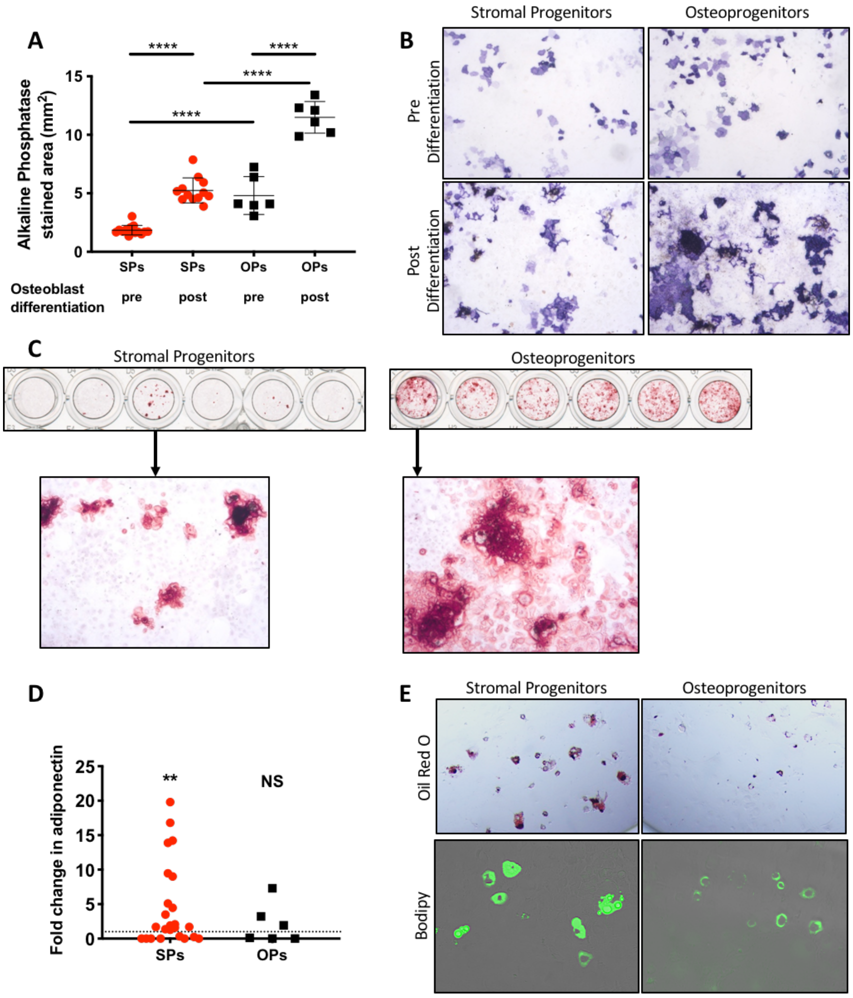

Supplementary Figure 3: Characterisation of osteoprogenitors (ALCAM+Sca1-) and stromal progenitor cells (Sca1+ALCAM-)

Stromal progenitor cells (Sca1+ ALCAM-) and osteoprogenitors (ALCAM+, Sca1-) were sorted from the bone lining of hindlimb long bones of healthy mice and cultured in control, osteogenic or adipogenic media.

(A) Alkaline phosphatase activity was quantitated pre and post culture in osteogenic media. ****$p<0.0001$, data represent mean ± s.d. One-way ANOVA, Sidak's multiple comparison test. SP; stromal progenitor. OP; osteoprogenitor

(B) Cells were stained for alkaline phosphatase, using fast blue RR salt staining, pre and post culture in osteogenic media.

(C) Cells were stained for alizarin red following culture in osteogenic media.

(D) Adiponectin, expressed relative to undifferentiated (day 3) conditions was measured in culture media following 21 days culture in adipogenic media. **$p<0.01$ as compared to day 3 undifferentiated conditioned media. Sidak's multiple comparison test. SP; stromal progenitor. OP; osteoprogenitor

(E) Adipocytes were visualized using Oil Red O and Bodipy following culture in adipogenic media.

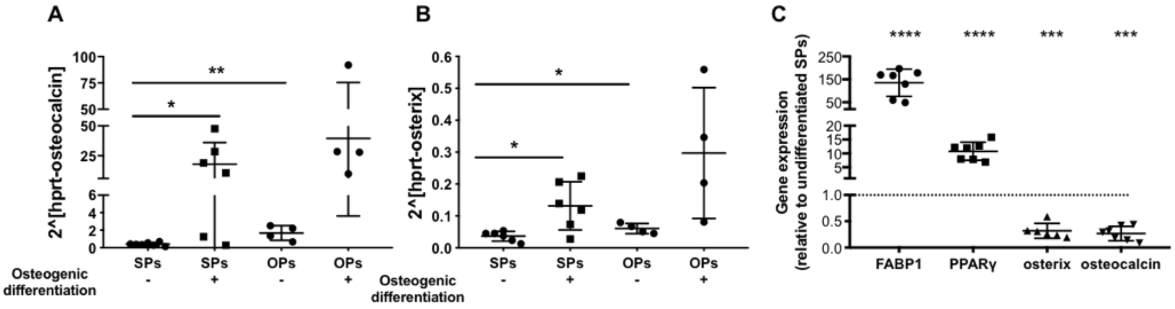

Supplementary Figure 4: Gene expression in osteoprogenitors (ALCAM+Sca1-) and stromal progenitor cells (Sca1+ALCAM-)

In separate experiments, stromal progenitor cells (Sca1+ ALCAM-) and osteoprogenitors (ALCAM+, Sca1-) were sorted from the bone lining of hindlimb long bones of healthy mice and cultured in control, osteogenic or adipogenic media.

(A) Expression of *osteocalcin* in SPs and OPs after culture in osteogenic or control media.

(B) Expression of *osterix* in SPs and OPs after culture in osteogenic or control media.

(C) Expression of *FABP1*, *PPAR-γ, osterix* and *osteocalcin* in SPs after culture in adipogenic or control media, expressed relative to control (undifferentiated) conditions.

SP; stromal progenitor, OP; osteoprogenitor, data represent mean ± s.d. *$p<0.05$, **$p<0.01$, ***$p<0.001$, t-test.

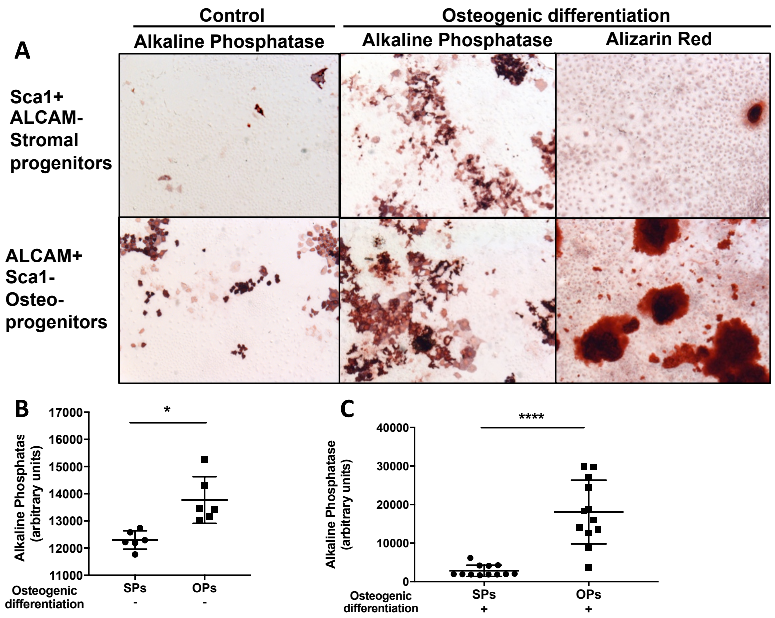

Supplementary Figure 5: Osteogenic differentiation of osteoprogenitors (ALCAM+Sca1-) and stromal progenitor cells (Sca1+ALCAM-) in myeloma-bearing mice

Stromal progenitors and osteoprogenitors sorted from the bone lining of hindlimb long bones of myeloma-bearing mice, and cultured in control or osteogenic media.

(A) Cells were stained for alkaline phosphatase (using fast violet B salt staining) or alizarin red.

(B) Alkaline phosphatase activity in stromal progenitors (SPs) and osteoprogenitors (OPs) sorted from myeloma-bearing mice, after culture to confluency (7 days).

(C) As (H), after further differentiation in osteogenic media (7 days).

SP; stromal progenitor, OP; osteoprogenitor, data represent mean ± s.d. *p<0.05, ***p<0.001, t-test.

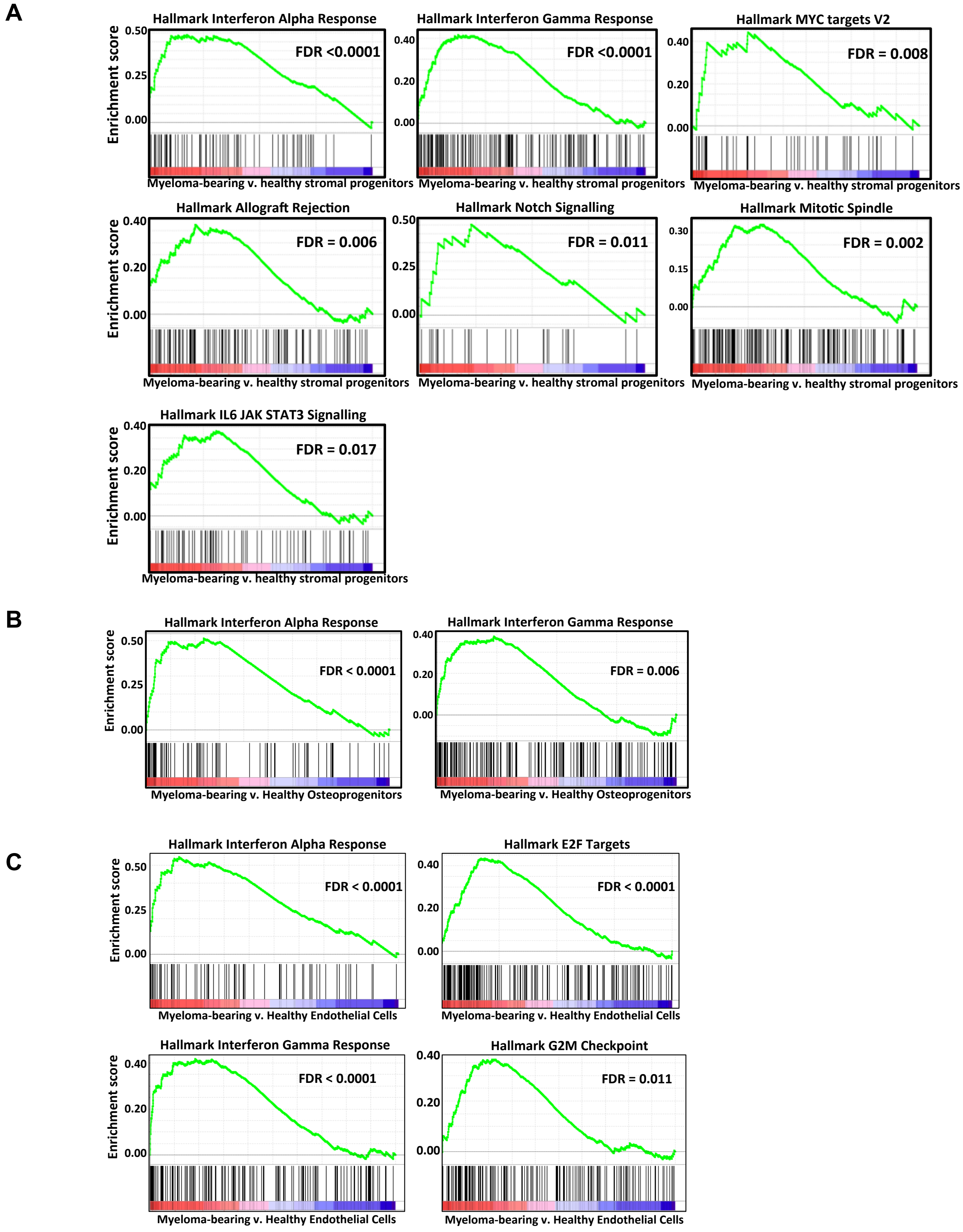

Supplementary Figure 6: Screening GSEA analysis of gene expression in bone lining niche components from myeloma-bearing and healthy mice using MSigDB Hallmark gene sets[57].

Hallmark gene sets are shown if they are positively enriched in myeloma-bearing mice with FDR<0.05.

(A) Sca1+ALCAM- stromal progenitors

(B) ALCAM+Sca1- osteoprogenitors

(C) CD31+ endothelial cells

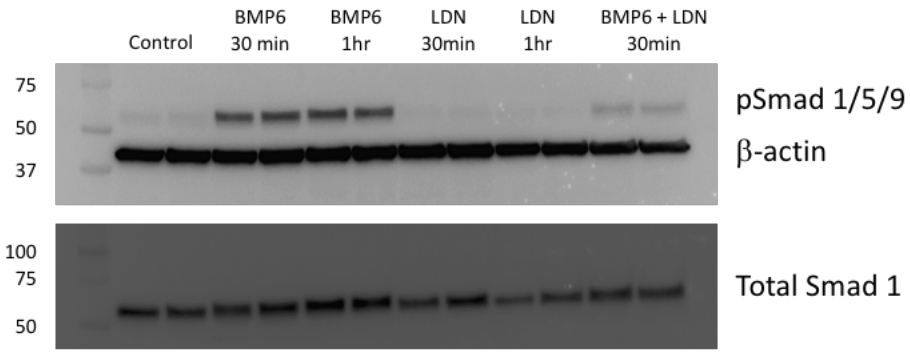

Supplementary Figure 7: Phosphorylated Smad1/5/9: Total Smad1 ratio in response to BMP6 or LDN treatment of 2T3 cells

Western blot demonstrating treatment of pre-osteoblast 2T3 cells with human BMP6 (200ng/ml) and LDN (100nM) for time as marked. Source data are provided as a Source Data file. Ladder size indicated in kDa.

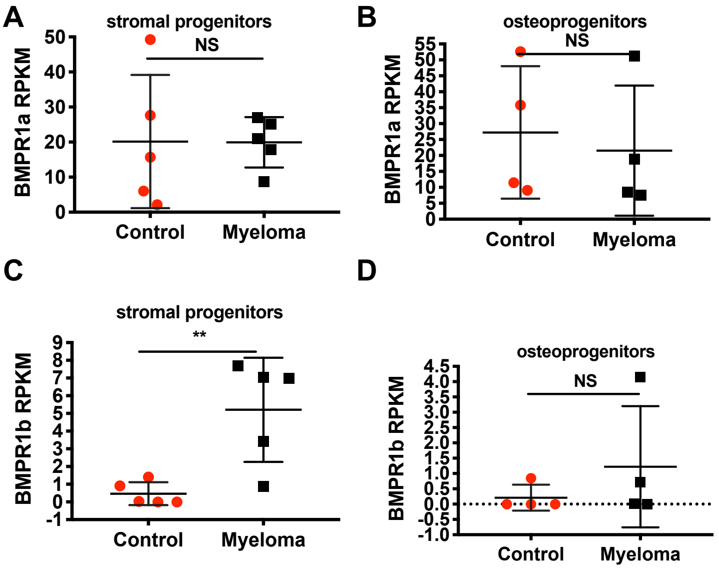

Supplementary Figure 8: BMPR1a and BMPR1b expression in stromal progenitors and osteoprogenitors

(A) BMPR1a expression in stromal progenitors

(B) BMPR1a expression in osteoprogenitors

(C) BMPR1a expression in stromal progenitors

(D) BMPR1a expression in osteoprogenitors

Data represent mean ± s.d.** $p<0.01$, t-test

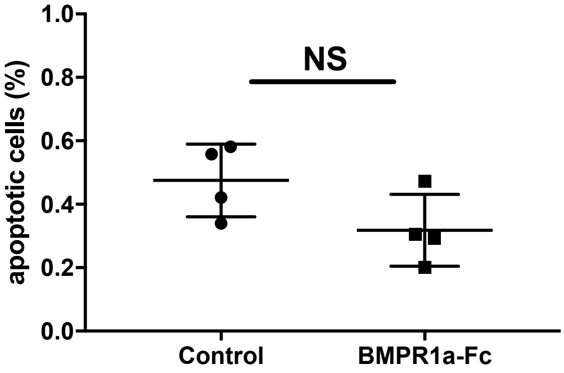

Supplementary Figure 9: Effect of BMPR1a-Fc on 5TGM1 myeloma cell apoptosis in vivo

Apoptotic myeloma cells (TUNEL+/GFP+) are expressed as a proportion of total myeloma cells. Data represent mean ± s.d. t-test

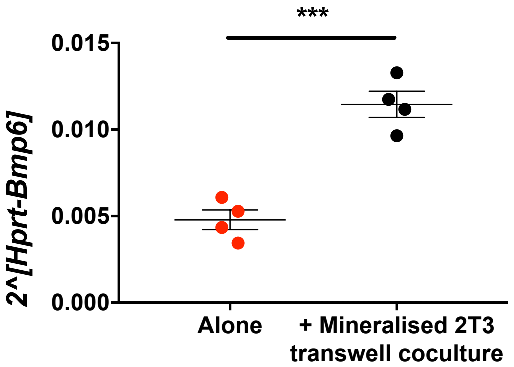

Supplementary Figure 10: 5TGM1 BMP6 expression is increased on 24h transwell co-culture with mineralized 2T3 cells

(n=4 replicates)

5TGM1 cell BMP2/4/6 gene expression analysis demonstrated only BMP6 is expressed, therefore BMP2 and BMP4 not shown. Data represent mean ± s.d. ***$p<0.001$, t-test.

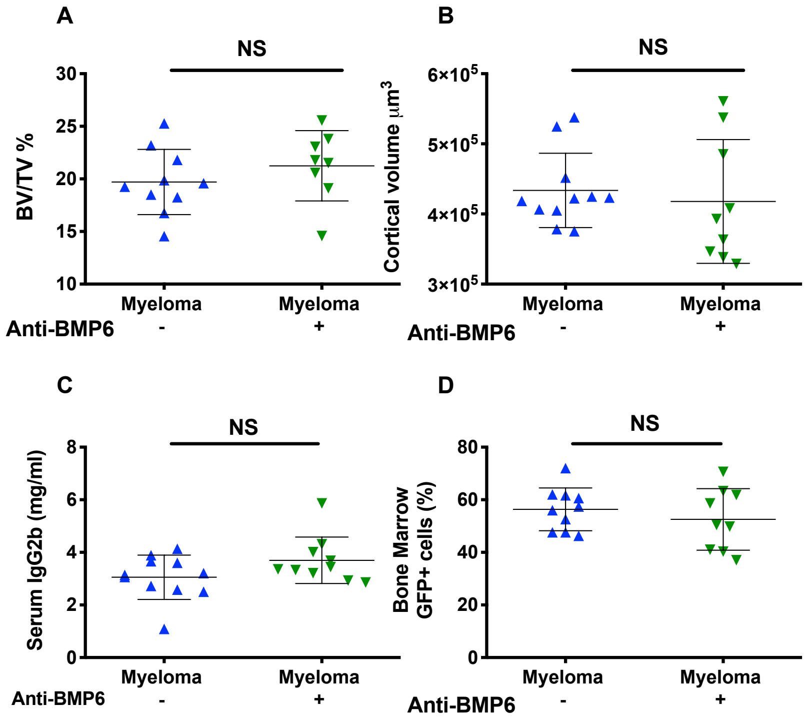

Supplementary Figure 11: Specific blockade of BMP6 with Anti-BMP6 *in vivo* has no effect on trabecular or cortical bone mass or tumour burden (5TGM1 model).

n = 11/Isotype control group, 10/Anti-BMP6 group. Missing data values: see methods. Reconstructed tibial metaphyseal images analysed using Bruker CTAn software to generate: Data represent mean ± s.d., t-test

(A) Total BV/TV (bone volume / total volume)

(B) Cortical volume in μm^3

(C) 5TGM1-GFP myeloma burden quantification using serum IgG2b level (mg/ml)

(D) 5TGM1-GFP myeloma burden quantification using bone marrow GFP+ cell percentage

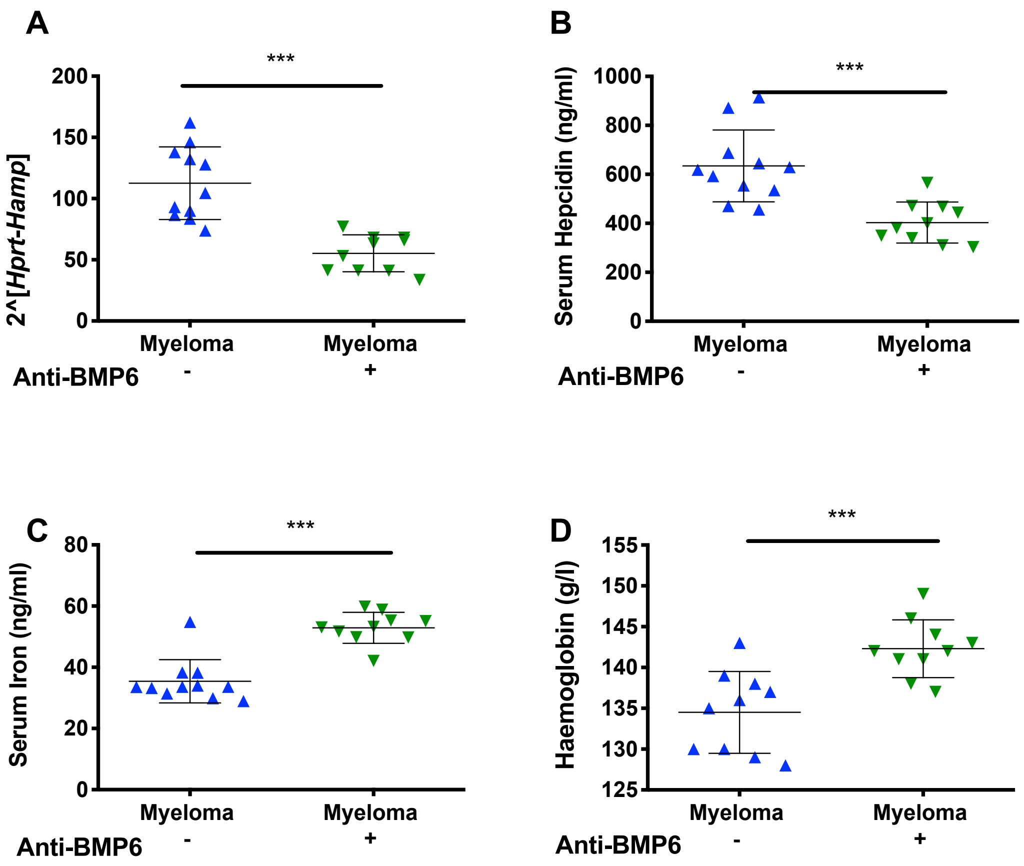

Supplementary Figure 12: Specific blockade of BMP6 with Anti-BMP6 *in vivo* reduces liver hepcidin expression and serum hepcidin, and increases serum iron and haemoglobin level (5TGM1 model).

n = 11/Isotype control group, 10/Anti-BMP6 group. Missing data values: see supplementary methods.

(A) Liver Hepcidin (Hamp) gene expression (2−ΔCT)

(B) Serum hepcidin concentration (ng/ml)

(C) Serum iron (ng/ml)

(D) Haemoglobin (g/L)

Data represent mean ± s.d. ***p<0.001, t-test.

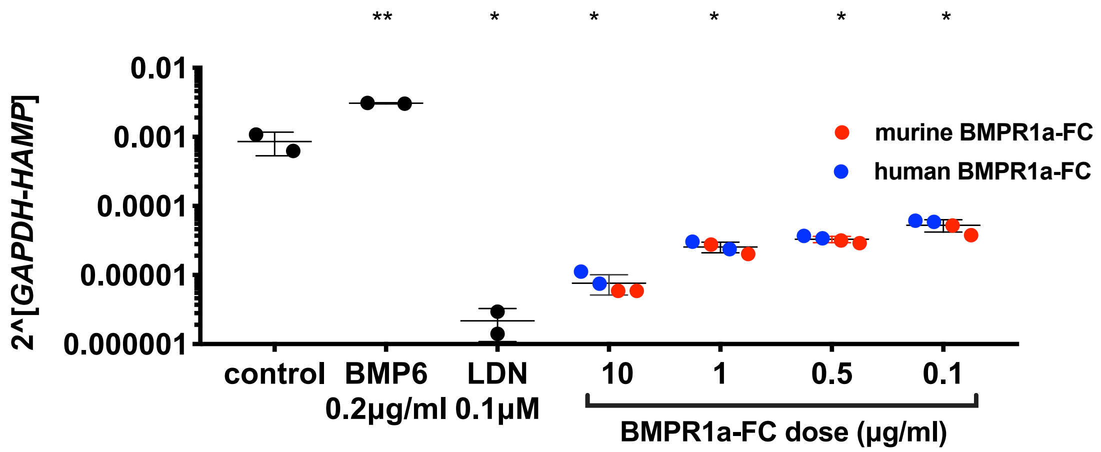

Supplementary Figure 13: *In vitro* suppression of *HAMP* gene expression ($2^{-\Delta CT}$) in HuH7 human hepatoma cells by BMPR1a-Fc.

Dose response curve comparing potency of BMPR1a-FC to 0.1µM LDN-193189. n = 4 replicates, 2 using human BMPR1a-FC, 2 using murine BMPR1a-FC. Data represent mean ± s.d. *p<0.05, **p<0.01, One-way ANOVA.

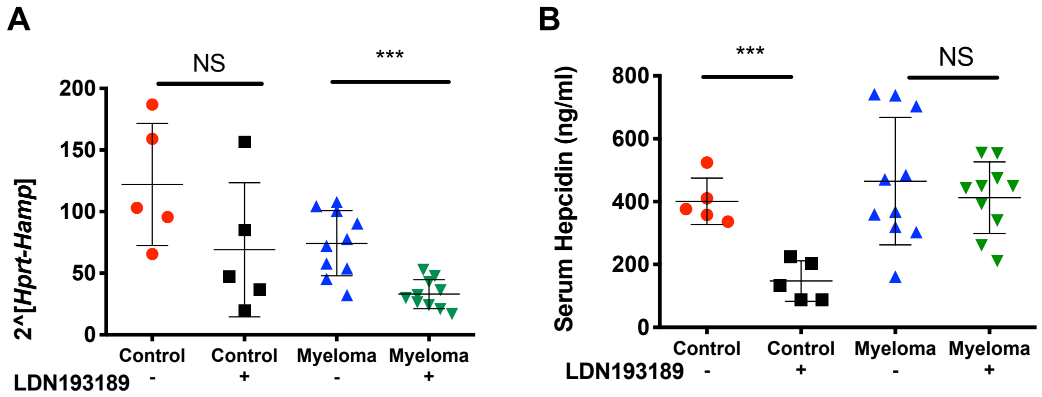

Supplementary Figure 14: Indices of anaemia in myeloma-bearing mice following treatment with LDN193189 (5TGM1 model)

n = 10/control group, 20/myeloma group, total n = 60 for microCT-derived indices; n = 5/control group, 10/myeloma group, total n = 30 for other indices. Missing data values: see supplementary methods.

(A) Liver *Hamp* gene expression ($2^{-\Delta CT}$)

(B) Serum hepcidin concentration (ng/ml)

Data represent mean ± s.d. ***p<0.001, t-test.

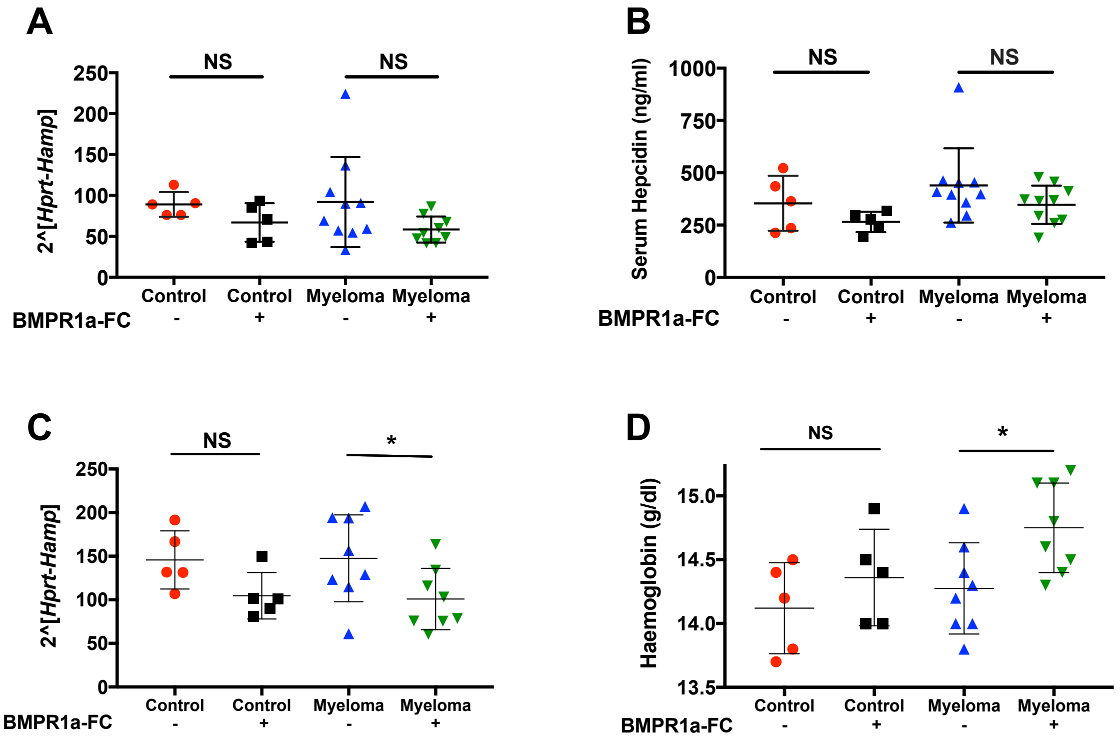

Supplementary Figure 15: Indices of anaemia in myeloma-bearing mice (5TGM1 and JJN-3 models) following treatment with BMPR1a-Fc

(5TGM1 model; n = 5/ control group, 10/ myeloma group, total n = 30.

JJN-3 model; n=5/control group, 8/myeloma group, total n=26)

(A) Liver Hepcidin *(Hamp)* gene expression ($2^{-\Delta CT}$) in healthy or 5TGM1 myeloma-bearing mice treated with BMPR1a-FC or vehicle.

(B) Serum Hepcidin concentration (ng/ml) in healthy or 5TGM1 myeloma-bearing mice treated with BMPR1a-FC or vehicle.

(C) Liver Hepcidin (*Hamp*) gene expression ($2^{-\Delta CT}$) in healthy or JJN-3 myeloma-bearing mice treated with BMPR1a-FC or vehicle.

(D) Haemoglobin (g/L) in healthy or JJN-3 myeloma-bearing mice treated with BMPR1a-FC or vehicle.

Data represent mean ± s.d. *p<0.05, t-test

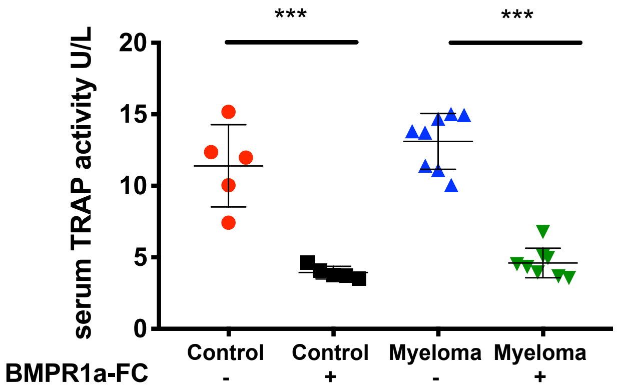

Supplementary Figure 16: Serum TRAP5b concentrations in JJN-3 myeloma-bearing mice following treatment with BMPR1a-Fc

n=5/control group, 8/myeloma group, total n=26). Serum TRAP5b activity (U/L) Data represent mean ± s.d.***p<0.001. t-test.

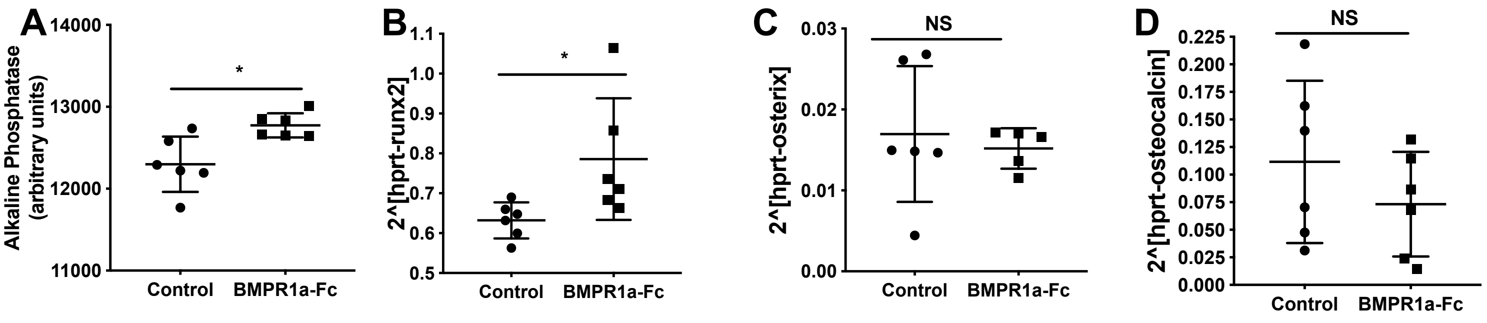

Supplementary Figure 17: Characterization of cultured stromal progenitors (ALCAM-, Sca1+) isolated from 5TGM1 myeloma-bearing mice treated with BMPR1a-Fc

n=6/group total n=26

(A) Alkaline phosphatase (arbitrary units)

(B) *Runx2* gene expression

(C) *Osterix* gene expression

(D) *Osteocalcin* gene expression

Data represent mean ± s.d. *p<0.05, t-test

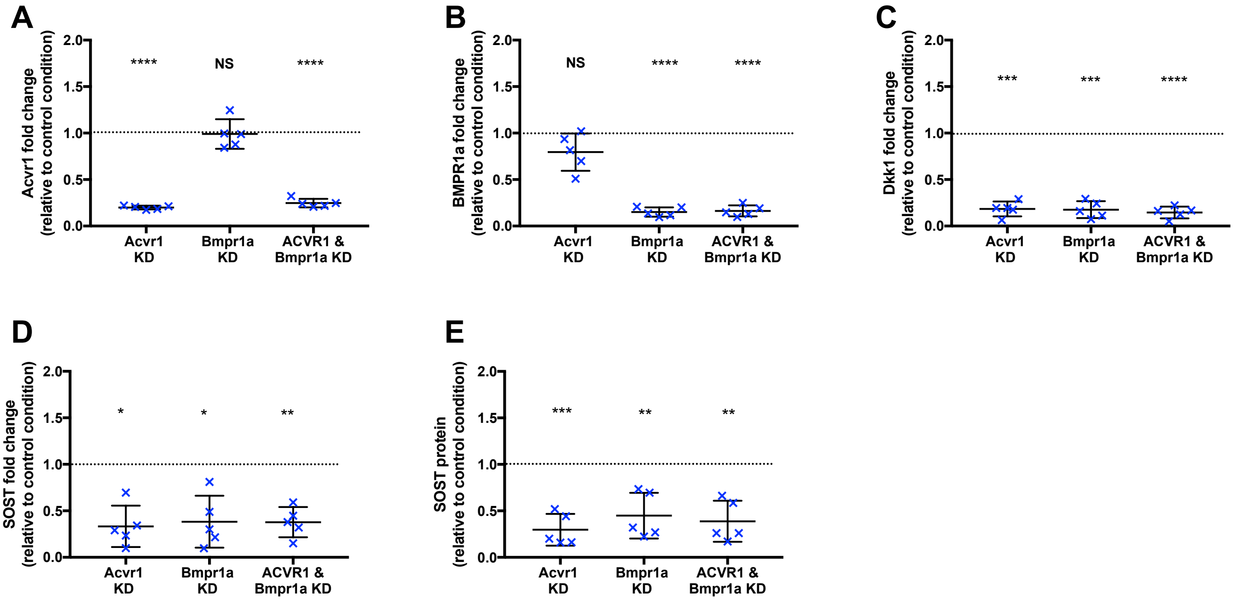

Supplementary Figure 18: Effect of single and double knockdown (KD) of Acvr1 and BMPR1a in osteosarcoma cells (UMR106.01)

(A) *Acvr1* gene expression

(B) *BMPR1a* gene expression

(C) *Dkk1* gene expression

(D) *SOST* gene expression

(E) SOST protein expression

Data represent mean ± s.d. p<0.05, **p<0.01, ***p<0.001, ****p<0.0001 as compared to control, One-way ANOVA

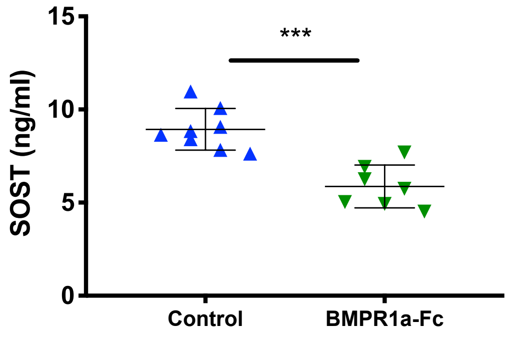

Supplementary Figure 19: Effect of BMPR1a-Fc on SOST in bone marrow plasma of JJN-3 myeloma-bearing mice

SOST protein expression (ng/ml). Data represent mean ± s.d. ***p<0.001, t-test.

SUPPLEMENTARY TABLES

	5TGM1 myeloma cells	CD31+ Endothelial cells	Sca1+ ALCAM- CD31- Stromal progenitors	ALCAM+ Sca1- CD31- Osteoprogenitors
Uniquely expressed	CD45 (Ptprc)<br>CD48 (Slamf2)<br>CD319 (Slamf7)<br>Igj	P-selectin (Selp)<br>VE-cadherin (Cadh5)<br>Angpt1 receptor (Tek)<br>CD31 (Pecam1)		ALCAM (CD166)<br>Bmp7<br>Dkk1<br>Osterix (Sp7)<br>N-cadherin (Cdh2)
Enriched	Cxcr4	Vcam1<br>Endoglin (Eng)<br>Fabp4<br>Ly6a (Sca1)<br>Cxcl12	Ly6a (Sca1)<br>Lepr<br>Pdgrfa<br>Pdgfrb<br>Prrx1<br>Cxcl12	Osteocalcin (Bglap)<br>Sox9<br>Col1a1<br>Runx2<br>Alpl<br>Cxcl12<br>Lepr<br>Pdgfrb
Some expression but less than another cell type	Alpl<br>Pecam1		Sox9<br>Col1a1<br>Runx2<br>Fabp4<br>Osteocalcin (Bglap)<br>Alpl	Pdgfra<br>Ly6a (Sca1)<br>Prrx1<br>Vcam1<br>Fabp4

Supplementary Table 1:

Gene expression markers used to phenotype bone lining cell compartments.

Nakamura *el al*[47] demonstrated that Alcam-Sca1+ stromal progenitors had the potential to differentiate down osteogenic or adipogenic pathways, although our gene expression analysis confirmed early osteogenic differentiation was present at least in some cells in 100-cell subsets. It is likely to contain a range of early commitment stages. The Alcam+Sca1- fraction however reportedly retained poor ability to pursue adipogenic differentiation[47] and our analysis confirmed a range of osteoblastic markers uniquely present or enriched in this population (Bglap, Col1a1, Sox9, Cdh2, Bmp7, Sp7).

Gene Set Name	NES	FDR
HALLMARK_EPITHELIAL_MESENCHYMAL_TRANSITION	2.32	<0.0001
HALLMARK_KRAS_SIGNALING_UP	1.84	0.0021
HALLMARK_ANGIOGENESIS	1.91	0.0021
HALLMARK_COAGULATION	1.77	0.0033
REACTOME_COLLAGEN_FORMATION	1.95	0.0178
REACTOME_NCAM1_INTERACTIONS	2.01	0.0193
REACTOME_EXTRACELLULAR_MATRIX_ORGANIZATION	1.96	0.0250
REACTOME_NCAM_SIGNALING_FOR_NEURITE_OUT_GROWTH	1.90	0.0287
HALLMARK_UV_RESPONSE_DN	1.52	0.0456
HALLMARK_GLYCOLYSIS	1.50	0.0492
HALLMARK_HYPOXIA	1.53	0.0494
HALLMARK_MYOGENESIS	1.54	0.0535
HALLMARK_ALLOGRAFT_REJECTION	1.42	0.1017
REACTOME_RESPONSE_TO_ELEVATED_PLATELET_CYTOSOLIC_CA2_	1.78	0.1033
HALLMARK_APICAL_JUNCTION	1.36	0.1659
REACTOME_A_TETRASACCHARIDE_LINKER_SEQUENCE_IS_ REQUIRED_FOR_GAG_SYNTHESIS	1.71	0.1913
REACTOME_INTEGRIN_CELL_SURFACE_INTERACTIONS	1.69	0.2036
HALLMARK_CHOLESTEROL_HOMEOSTASIS	1.31	0.2325

Supplementary Table 2:

Hallmark and Reactome GSEA MSigDB Gene sets that were enriched in bulk, non-bone lining adherent 5TGM1 myeloma cells from vehicle-treated mice compared to mice treated with LDN193189 (FDR<0.25). Note no gene sets were enriched in myeloma cells from LDN-treated mice.

Cell type	Gene Set Name	NES	FDR
**Stromal progenitors**	HALLMARK_G2M_CHECKPOINT	1.0402256	0.7332428
**Stromal progenitors**	HALLMARK_E2F_TARGETS	1.0377486	0.6958631
**Stromal progenitors**	HALLMARK_MITOTIC_SPINDLE	0.8427662	0.99195504
**Osteoprogenitors**	HALLMARK_G2M_CHECKPOINT	1.2476387	0.5981149
**Osteoprogenitors**	HALLMARK_E2F_TARGETS	1.0063123	0.77475333
**Osteoprogenitors**	HALLMARK_MITOTIC_SPINDLE	0.9143548	0.9626063

Supplementary Table 3:

Gene set enrichment analysis of gene sets associated with cell cycle in stromal progenitors and osteoprogenitors from LDN-treated vs vehicle-treated myeloma-bearing mice showed no significant enrichment to suggested altered proliferation as a result of LDN treatment.

Patient sex, age	Clinical diagnosis	Previous treatments	Aspirate sample Plasma cell %
m, 74	Myeloma post induction	VCD induction	2
m, 68	New myeloma without bone lesions	Nil	10
m, 65	Myeloma, post high dose melphalan autograft	Induction + ASCT	2
m, 70	Myeloma extensively pretreated	RCD, 2x ASCT, R maintenance	10
m, 77	New myeloma without bone lesions	Nil	15
m, 75	New myeloma with bone lesions	Nil	20
m, 69	MGUS	Nil	10

Supplementary Table 4:

Patient characteristics associated with clinical samples used to generate BMSC cultures.

Diagnosis and plasma cell percentage (if relevant) of patient samples used to generate BMSC cultures used to measure gene expression changes in response to LDN (n=7) or BMPR1a treatment (n=4, same patients) (Figure 6H). VCD = Bortezomib, Cyclophosphamide and Dexamethasone treatment; ASCT = autologous stem cell transplant; RCD = Lenalidomide, Cyclophosphamide and Dexamethasone treatment; R = Lenalidomide monotherapy.

Cellular Target	Fluorochrome	Source	Clone	Working Dilution
CD45	AF700	eBioscience Cat# 56-0451-82	30-F11	1/50
Ter119	BV650	Biolegend Cat# 116235	TER-119	1/50
CD31	APC	eBioscience Cat# 17-0311-82	390	1/800
Sca-1	PeCy7	Biolegend Cat# 122514	E13-161.7	1/200
ALCAM (CD166)	PE	R&D Systems Cat# FAB1172P	Polyclonal Goat IgG (FAB1172P)	1/10
Dead cells	7AAD	eBioscience Cat# 00-6993-50		1/100

Supplementary Table 5:

Antibody panel used for FACS sorting of bone lining niche cells

**Species**	**Gene**	**Probe**
Human	*GAPDH*	Hs999905
	HAMP	Hs00221783
	POLR2A	Hs00172187
	DKK1	Hs00183740
	SMAD6	Hs00178579
	ATOH8	Hs01031629
	SMAD7	Hs00998193
	ID1	Hs03676575
	ID3	Hs00171409
Mouse	*Hprt*	Mm03024075
	Hamp	Mm04231240
	Smad6	Mm00484738
Rat	*Hprt*	Rn01527840
	Acvr1	Rn01449846
	Bmpr1a	Rn01450292
	Sost	Rn00577971
	Dkk1	Rn01501537

Supplementary Table 6:

Taqman probes used
