# Supplementary material for: Transcriptomic profiling of the myeloma bone-lining niche reveals BMP signalling inhibition to improve bone disease
Source: Nat Commun. 2019 Oct 4;10:4533. doi: 10.1038/s41467-019-12296-1 (PMC6778199; doi:10.1038/s41467-019-12296-1)
Supplement: Supplementary file 2 — Description of Additional Supplementary Files [file 41467_2019_12296_MOESM2_ESM.pdf]

Description of Additional Supplementary Files

File Name: Supplementary Data 1
Description:
Bone homeostasis gene sets.
Lists of genes included in each gene set:
(A) Bone loss
(B) Bone formation
(C) Bone remodeling (original)
(D) BMP signaling pathway
(E) Bone remodeling with BMP pathway supplementation

File Name: Supplementary Data 2
Description:
Significantly enriched REACTOME gene sets.
Lists of genes included in REACTOME gene sets that were significantly enriched in stromal progenitors from LDN-treated myeloma-bearing mice:
(A) Nuclear Receptor Transcription pathway
(B) Extracellular Matrix Formation
(C) Collagen Formation
(D) NCAM1 Interactions
