# Supplementary material for: Transcriptomic profiling of the myeloma bone-lining niche reveals BMP signalling inhibition to improve bone disease
Source: Nat Commun. 2019 Oct 4;10:4533. doi: 10.1038/s41467-019-12296-1 (PMC6778199; doi:10.1038/s41467-019-12296-1)
Supplement: Supplementary file 5 — Reporting Summary [file 41467_2019_12296_MOESM5_ESM.pdf]

nature research

Corresponding author(s): Claire Edwards

Last updated by author(s): Sep 11, 2019

Reporting Summary

Nature Research wishes to improve the reproducibility of the work that we publish. This form provides structure for consistency and transparency in reporting. For further information on Nature Research policies, see Authors & Referees and the Editorial Policy Checklist.

Statistics

For all statistical analyses, confirm that the following items are present in the figure legend, table legend, main text, or Methods section.

n/a	Confirmed	
☐	☒	The exact sample size (*n*) for each experimental group/condition, given as a discrete number and unit of measurement
☐	☒	A statement on whether measurements were taken from distinct samples or whether the same sample was measured repeatedly
☐	☒	The statistical test(s) used AND whether they are one- or two-sided<br>*Only common tests should be described solely by name; describe more complex techniques in the Methods section.*
☒	☐	A description of all covariates tested
☐	☒	A description of any assumptions or corrections, such as tests of normality and adjustment for multiple comparisons
☐	☒	A full description of the statistical parameters including central tendency (e.g. means) or other basic estimates (e.g. regression coefficient) AND variation (e.g. standard deviation) or associated estimates of uncertainty (e.g. confidence intervals)
☐	☒	For null hypothesis testing, the test statistic (e.g. *F*, *t*, *r*) with confidence intervals, effect sizes, degrees of freedom and *P* value noted<br>*Give P values as exact values whenever suitable.*
☒	☐	For Bayesian analysis, information on the choice of priors and Markov chain Monte Carlo settings
☒	☐	For hierarchical and complex designs, identification of the appropriate level for tests and full reporting of outcomes
☒	☐	Estimates of effect sizes (e.g. Cohen's *d*, Pearson's *r*), indicating how they were calculated

Our web collection on statistics for biologists contains articles on many of the points above.

Software and code

Policy information about availability of computer code

Data collection	N/A
Data analysis	N/A

For manuscripts utilizing custom algorithms or software that are central to the research but not yet described in published literature, software must be made available to editors/reviewers. We strongly encourage code deposition in a community repository (e.g. GitHub). See the Nature Research guidelines for submitting code & software for further information.

Data

Policy information about availability of data

All manuscripts must include a data availability statement. This statement should provide the following information, where applicable:

- Accession codes, unique identifiers, or web links for publicly available datasets
- A list of figures that have associated raw data
- A description of any restrictions on data availability

RNA sequencing data have been deposited in NCBI's Gene Expression Omnibus and are accessible through accession code GSE135786 (https://www.ncbi.nlm.nih.gov/geo/query/acc.cgi?acc=GSE135786) . Other data are available from the authors upon reasonable request.

Field-specific reporting

Please select the one below that is the best fit for your research. If you are not sure, read the appropriate sections before making your selection.

☒ Life sciences ☐ Behavioural & social sciences ☐ Ecological, evolutionary & environmental sciences

For a reference copy of the document with all sections, see nature.com/documents/nr-reporting-summary-flat.pdf

Life sciences study design

All studies must disclose on these points even when the disclosure is negative.

Sample size	Taking into consideration '3Rs' guidance in use of animals for scientific research, standard sample size for pharmacological intervention studies was n = 30 mice, 5 per control group, 10 per myeloma-bearing group (power analysis indicated that n=10 mice per group would be required to detect a significant change in tumour burden).
Data exclusions	Figures 2, 4, 5 and supplementary Figure 11 microCT and histomorphometric data have missing data values (noted in each legend) relative to total experimental n. This is due to broken/damaged bones at harvest, especially likely if severe bone disease is present. In Fig. 2L two GFP percentage data values are missing from the myeloma + LDN cohort due to sample contamination.
Replication	Experiments were reproduced with attempts at replication successful
Randomization	Mice were randomly allocated into groups prior to the experiment
Blinding	Investigators were blinded to mouse treatment during analysis

Reporting for specific materials, systems and methods

We require information from authors about some types of materials, experimental systems and methods used in many studies. Here, indicate whether each material, system or method listed is relevant to your study. If you are not sure if a list item applies to your research, read the appropriate section before selecting a response.

Materials & experimental systems

n/a	Involved in the study	
☐	☒	Antibodies
☐	☒	Eukaryotic cell lines
☐	☐	Palaeontology
☐	☒	Animals and other organisms
☐	☒	Human research participants
☒	☐	Clinical data

Methods

n/a	Involved in the study	
☒	☐	ChIP-seq
☐	☒	Flow cytometry
☒	☐	MRI-based neuroimaging

Antibodies

Antibodies used	Details of antibodies used are in Table S7
Validation	Antibodies were fully validated by the supplier

Eukaryotic cell lines

Policy information about cell lines

Cell line source(s)	Huh7 cells (an immortalized human hepatoma cell line) were a gift of Prof Persephone Borrow, University of Oxford. UMR-106 cell line (ECACC, 90111314) was a gift of Dr Philippa Hulley, University of Oxford. JJN3 (DSMZ, ACC 541) and MM1-S (ATCC CRL-2974) cell lines were a gift of Prof Udo Oppermann, University of Oxford. JJN3 myeloma cells (DSMZ, ACC 541) used for in vivo studies were a gift of Dr. Shelly Lawson, University of Sheffield. 2T3 mouse preosteoblasts30 were a kind gift from Dr. Steve Harris, University of Texas Health Science Center at San Antonio and HS5 human stromal cell line were obtained from ATCC (CRL-11882). 5TGM1 and 5TGM1-GFP murine myeloma cells59 were a kind gift from Prof. Gregory Mundy, University of Texas Health Science Center at San Antonio.
Authentication	5TGM1 murine cell line has no standard authentication available. Authentication based upon function (induction of myeloma in vivo) and production of IgG2bkappa. JJN-3 and MM1.S cells were authenticated by STR. 2T3, HS5, Huh7 and UMR-106 cell lines were not formally authenticated, but cell lines were obtained from trusted sources and behaved throughout experiments according to expectations - eg expressing cell-type specific genes and exhibiting cell-type specific function.
Mycoplasma contamination	All cell lines routinely tested negative for mycoplasma
Commonly misidentified lines (See ICLAC register)	*Name any commonly misidentified cell lines used in the study and provide a rationale for their use.*

Palaeontology

Specimen provenance	*Provide provenance information for specimens and describe permits that were obtained for the work (including the name of the issuing authority, the date of issue, and any identifying information).*
Specimen deposition	*Indicate where the specimens have been deposited to permit free access by other researchers.*
Dating methods	*If new dates are provided, describe how they were obtained (e.g. collection, storage, sample pretreatment and measurement), where they were obtained (i.e. lab name), the calibration program and the protocol for quality assurance OR state that no new dates are provided.*

☐ Tick this box to confirm that the raw and calibrated dates are available in the paper or in Supplementary Information.

Animals and other organisms

Policy information about studies involving animals; ARRIVE guidelines recommended for reporting animal research

Laboratory animals	Species: mouse, Strain: KaLwRij, NSG, Age, 12 weeks
Wild animals	*Provide details on animals observed in or captured in the field; report species, sex and age where possible. Describe how animals were caught and transported and what happened to captive animals after the study (if killed, explain why and describe method; if released, say where and when) OR state that the study did not involve wild animals.*
Field-collected samples	*For laboratory work with field-collected samples, describe all relevant parameters such as housing, maintenance, temperature, photoperiod and end-of-experiment protocol OR state that the study did not involve samples collected from the field.*
Ethics oversight	Animal experiments were undertaken under UK Home Office Project License 30/2996

Note that full information on the approval of the study protocol must also be provided in the manuscript.

Human research participants

Policy information about studies involving human research participants

Population characteristics	Patients with or under investigation for myeloma at Oxford University Hospitals NHS Trust, consenting for the use of their bone marrow aspirate samples for research purposes.
Recruitment	All patients with or under investigation for myeloma undergoing bone marrow aspiration, are given the opportunity to consent for donating excess bone marrow cells, plus accompanying anonymised clinical data collected, to research projects under the oversight of the Oxford Radcliffe Biobanking (ORB) program.
Ethics oversight	This work was approved by Oxford Clinical Research Ethics Committee (09/H0606/5 project 13/A238).

Note that full information on the approval of the study protocol must also be provided in the manuscript.

Flow Cytometry

Plots

Confirm that:

☒ The axis labels state the marker and fluorochrome used (e.g. CD4-FITC).

☒ The axis scales are clearly visible. Include numbers along axes only for bottom left plot of group (a 'group' is an analysis of identical markers).

☒ All plots are contour plots with outliers or pseudocolor plots.

☒ A numerical value for number of cells or percentage (with statistics) is provided.

Methodology

Sample preparation	Bilateral femora and tibiae were cleaned, crushed, repeatedly washed to remove all marrow, then minced finely with scissors. Fragments were incubated in sterile 37°c 3mg/ml Type 1 Collagenase (Worthington) in PBS (5% fetal calf serum (FCS), 200U/ml DNAse1) on rotation for 30 minutes to allow matrix digestion, after which the released cell suspension was filtered, washed and stored on ice in PBS (5%FCS 2mM EDTA). Bone fragments were subjected to a second 30-minute incubation in fresh Collagenase solution. Cells released from the second incubation were added to those from the first, and all cells treated with FC block then stained with antibody cocktail (antibodies detailed in Supplementary Table S7). They were sorted as per main methods (FACS plots shown in Supplementary Figure S1), with FMO controls used to determine gates.
Instrument	BD FACSAria III Cell Sorter (BD Biosciences)
Software	FACSDiva v8.0.1 and Flowjo v10

Cell population abundance	Representative plots of the abundance of the 4 populations sorted from bone lining cells (GFP+ myeloma cells, CD31+ cells, ALCAM+Sca1- cells and Sca1+ALCAM- cells), as a proportion of the live cell gate, have been added to Figure 1A and Figure S1. The GFP+ myeloma cell abundance in the bone lining fraction varied depending on tumour burden. Purity checks of sorted populations were performed once per sorting session, and purity of sorted populations verified to be over 90%.
Gating strategy	Gating strategy is laid out in Figure S1. Parent gates for each plot are stated above each plot. Antibodies and sources are detailed in table S7. All gate positions were determined using an FMO (Fluorescence minus one) control for each fluorochrome.

☒ Tick this box to confirm that a figure exemplifying the gating strategy is provided in the Supplementary Information.
